# *SELAM*: selective ECC-based lightweight authentication for the internet of medical things

**DOI:** 10.1038/s41598-026-53641-x

**Published:** 2026-07-03

**Authors:** Azlina Ahmadi Julaihi, Md. Asri Ngadi, Raja Zahilah, Adnan Shahid Khan, Irshad Ahmed Abbasi, Abdulmonem Alshahrani, Eatedal Alabdulkreem

**Affiliations:** 1 Faculty of Computing, Universiti Teknologi Malaysia, Skudai, Johor Bahru, 81310 Johor Malaysia; 2 Faculty of Computer Science and Information Technology, Universiti Malaysia Sarawak, Kota Samarahan, 94300 Malaysia; 3 Department of Computer Science and Artificial Intelligence, College of Computing and Information Technology, University of Bisha, Bisha, 67714 Saudi Arabia; 4 Department of Informatics and Computer Systems, King Khalid University, Abha, 61421 Saudi Arabia; 5 Department of Computer Sciences, College of Computer and Information Sciences, Princess Nourah bint Abdulrahman University, P.O. Box 84428, Riyadh, 11671 Saudi Arabia

**Keywords:** Authentication, Elliptic curve cryptography (ECC), Internet of medical things (IoMT), Lightweight multi-factor authentication, Man-in-the-middle (MitM), Mutual authentication, Replay, Impersonation, BAN logic, Engineering, Mathematics and computing

## Abstract

Secure authentication in the Internet of Medical Things (IoMT) must ensure strong security while maintaining minimal computational overhead, especially for resource-constrained medical devices. This study introduces SELAM, a lightweight multifactor authentication framework optimized for critical IoMT applications. Unlike traditional designs, SELAM selectively confines elliptic-curve cryptography (ECC) to user/device registration, while relying on lightweight primitives (XOR, hashing/HMAC, and timestamp-freshness checks) in online operation to minimize runtime cost. The scheme is validated using the CICIoMT-2024 dataset through Python-based cryptographic simulation and ns-3 network emulation. Under standardized 16-byte online field accounting, SELAM reduces payload-only online communication to 6,144 bits/device versus 7,680 bits/device for a Heavy+Verify ECC baseline; in ns-3 header-inclusive accounting, this corresponds to 39,416 versus 45,703 bits/device at $\:N=136$. At 1 Mb/s, SELAM achieves 6.24 ms total per-device authentication overhead (communication + computation) compared to 31.27 ms for Heavy+Verify, while reducing online computation from 23.59 ms to 0.10 ms per device. Across cohort sizes $\:N=136$–$\:2000$ over 20 seeds (mean ± 95% CI), SELAM maintains attack-regime authentication success ratio (ASR) at 0.88–0.90 (baseline: 0.90–0.92), with protocol-level FAR=0 (no accepted replay/impersonation) and benign FRR=0 observed in PhaseLogs. Security analysis using BAN logic confirms mutual authentication and key confirmation on a fresh session key in Phases 4–5, with replay/impersonation resistance under the stated Dolev–Yao adversary and standard MAC/AEAD assumptions. The results indicate that confining ECC to registration preserves strong authentication while removing public-key operations from the performance-critical online path.

## Introduction

 The rapid advancement of the Internet of Things (IoT) has accelerated the adoption of intelligent systems in healthcare, giving rise to the Internet of Medical Things (IoMT). IoMT integrates wearable sensors, implantable devices, and remote monitoring systems with cloud-based services, enabling real-time patient monitoring, data-driven clinical decision-making, and improved healthcare delivery. According to recent projections, the IoMT market is expected to exceed USD 187 billion by 2028, driven by increasing demand for remote health monitoring and the growing prevalence of chronic diseases^1,2^. However, this proliferation of interconnected medical devices also introduces critical security and privacy challenges, as sensitive health information is frequently transmitted across resource-constrained environments. Ensuring the confidentiality, integrity, and authenticity of such data is therefore a paramount concern in the deployment of IoMT systems.

Authentication has emerged as a cornerstone security requirement for IoMT, acting as the first line of defense against unauthorized access, impersonation, and data tampering^3–6^. Conventional public-key authentication based on RSA and heavy handshake designs are often unsuitable for resource-constrained IoMT nodes due to computational and communication overhead^7,8^. In this context, Elliptic Curve Cryptography (ECC) has been widely adopted due to its ability to provide equivalent security with much smaller key sizes, thereby reducing computational and communication costs^9–11^. Despite these advantages, recent studies have highlighted a scalability bottleneck. For example, when the number of medical devices increases, the repeated invocation of ECC-based cryptographic operations introduces significant overhead, which degrades performance in large-scale IoMT environments^12–15^. This creates a critical research gap between the theoretical lightweightness of ECC and its real-world scalability in dynamic medical environments. Another issue is that many schemes treat all devices the same, ignoring the difference between critical and non-critical devices, which leads to unnecessary overhead and affects real-time responsiveness^15,16^.

A range of lightweight authentication schemes has been proposed in the literature to address IoT and IoMT security challenges^5,17,18^. Protocols such as^8,14,15,19,20^, and others introduce optimizations in terms of message exchanges, cryptographic complexity, and resistance against common attacks. While these approaches demonstrate improvements over conventional methods, they often rely on multiple rounds of ECC-based operations or additional cryptographic primitives, which collectively limit their applicability in resource-constrained IoMT deployments. Furthermore, most existing schemes do not adequately differentiate between the performance requirements of critical medical devices (e.g., ECG sensors, infusion pumps) and less time-sensitive devices, resulting in inefficiencies when scaled across heterogeneous environments.

Beyond ECC-heavy designs, several lightweight IoT/IoMT authentication protocols reduce runtime cost by relying primarily on hash/HMAC, XOR, or symmetric-key checks^3,10,15,19,21^. While these approaches can be efficient, they commonly assume pre-established shared secrets or stronger enrollment trust and may introduce additional coordination/state that becomes fragile under intermittent connectivity or at scale. Conversely, ECC-centric schemes preserve strong identity binding but often incur repeated online asymmetric verification per session, which dominates latency and energy consumption on constrained devices^8,9,11,22^. This motivates a design point that preserves ECC’s enrollment-time identity anchoring while eliminating online public-key operations. SELAM adopts this trade-off by confining ECC to one-time registration and using lightweight authenticated checks with KDF-based session-key derivation during online authentication.

Motivated by these limitations, this work proposes a Selective Elliptic Curve Lightweight Authentication Mechanism (SELAM) that streamlines authentication by integrating ECC with lightweight cryptographic operations such as hashing, XOR, and timestamp validation. Online session keys are derived via a key derivation function (KDF) from the registration seed and fresh nonces/timestamps, eliminating per-session ECC computations. The contributions to this work can be summarized as follows:

1. Lightweight Multi-Factor Authentication: We design SELAM, achieving mutual authentication while keeping the online path lightweight by using hash/HMAC, XOR, and timestamp freshness in all in-session phases.
2. Selective-ECC Placement: We confine ECC to one-time registration: device (Phase B1) and user (Phase 0) and replace per-session public-key work with lightweight primitives. Session-key derivation (Phase 4) employs a KDF, avoiding per-message/per-device ECC and directly addressing ECC-at-runtime scalability limits in IoMT.
3. Performance Evaluation and Benchmarking: Using the CICIoMT authentication logs, we implement a phase-accurate simulation of SELAM and report authentication delay, header-inclusive communication overhead, and per-phase cryptographic cost. Compared to a runtime-ECC Heavy+Verify (per-message/per-device ECC), SELAM lowers online computation and communication while preserving correctness.
4. Security Rationale (Informal): We provide an assumption-stated, phase-mapped analysis for replay, impersonation, and MitM threats. Resilience is argued but not formally proven, based on freshness checks (timestamps/nonces), context-bound MACs, and key-confirmation under standard assumptions (bounded clock skew, nonce uniqueness, secure long-term key storage).

To validate the proposed scheme, we simulate the full 10-phase authentication flow using the CICIoMT 2024 dataset^23^, which contains over 70,000 IoMT authentication logs, including both normal and malicious traffic. A custom cryptographic simulation engine was developed using real ECC, hash, and XOR operations to simulate the protocol’s 10 distinct authentication phases.

The remainder of this paper is structured as follows: Section II discusses related work and existing authentication schemes in IoT and IoMT. Section III presents the protocol model. Section IV details the proposed SELAM mechanism, including design principles and algorithmic workflow. Section V presents the simulation setup, performance evaluation, and benchmarking results. Section VI provides security analysis, while Section VII concludes the paper with key findings and directions for future work.

## Related work

Authentication protocols on the Internet of Medical Things (IoMT) have been widely studied due to the sensitivity of medical data and the constrained resources of devices. Several directions have been pursued in the literature, ranging from public-key-based authentication to lightweight symmetric and hybrid constructions. This section reviews representative approaches and highlights the design space that motivates the proposed selective-ECC model.

Public-key authentication is commonly realized using RSA-based signatures or key establishment, while symmetric primitives such as AES are used to protect data after a session key is established. RSA’s larger key sizes and computational cost can be burdensome for constrained IoMT nodes^17,24^. Elliptic Curve Cryptography (ECC) achieves comparable security with smaller public-key sizes, which can reduce both computation and communication overhead. For example, a 128-bit security level is typically associated with RSA-3072 or an elliptic curve of approximately 256 bits (e.g., P-256)^25,26^.

Recent authentication protocols for medical IoT have continued to refine lightweight designs while strengthening threat modeling and attack coverage. For instance, the scheme in^27^ emphasizes system-level resilience and protocol robustness under diverse attack conditions, while^28^ introduces enhanced security guarantees with more comprehensive adversary modeling for medical IoT environments. While these approaches improve security coverage, they still rely on either repeated online cryptographic verification or additional coordination mechanisms. In contrast, SELAM focuses specifically on eliminating online public-key operations for critical-device authentication while retaining ECC-based identity anchoring during registration.

### ECC-heavy online authentication

Numerous schemes in healthcare adopt ECC across multiple phases to provide strong security properties such as mutual authentication, anonymity, and resistance to impersonation and replay. Representative examples include ESEAP, which employs ECC in a multi-phase smart-card/healthcare handshake^22^; four-factor schemes that couple ECC with physical unclonable functions (PUFs) to raise assurance^29^; and ALMASH, which blends ECC with secret sharing to achieve anonymity-preserving mutual authentication^14^. These designs typically report sound security arguments and, in some cases, formal analyses; however, their recurrent ECC in login and online mutual-authentication phases leaves end-devices paying public-key costs per session, a clear bottleneck in dense clinical settings where many sensors authenticate concurrently.

### Group, batch, and blockchain/fog frameworks

The foundation of group-based selective authentication was first laid by^30^, who introduced a context-aware model where users authenticate selectively with a subset of IoT devices. Their approach reduced communication overhead by grouping authentication sessions but lacked advanced cryptographic mechanisms like ECC or robust session key management, limiting its security strength in real-time medical environments. Their work, while innovative, did not incorporate mutual authentication or group-based key derivation, making it unsuitable for high-assurance medical applications. More recent frameworks combine ECC with group processes and, in some cases, fog/blockchain coordination to curb server bottlenecks or enhance auditability^31–33^. To improve scalability, group-oriented and batch-based authentication frameworks have been introduced. For example in^16^, they proposed ID-LVEAS, a locally verifiable aggregated signature scheme enabling batch verification of aggregated medical data. Across these lines, however, end-devices still perform a public-key step during group joins or online handshakes before aggregation or batching pays, so the per-device compute and energy per session remain dominated by ECC, thus introduced coordination/storage overhead. The work of^34^ developed a blockchain-assisted privacy-aware framework for fog-enabled IoMT systems, while^32^ proposed a lightweight cross-hospital authentication scheme using ECC within a blockchain architecture. These frameworks improve auditability and distributed trust but increase architectural complexity and do not directly reduce the cryptographic overhead borne by medical devices.

### Lightweight and hash-centric schemes

Cluster-based designs, such as 3ECAP, bind users to sensor clusters and use three factors: password, biometrics via a fuzzy extractor, and a smart card^35^. 3ECAP avoids public-key work in the protocol and relies on hash and dissimilarity operations to keep device cost and message size low. The paper presents formal security in the ROR model, verification in ProVerif, and simulation in NS-3. Because its runtime path is hash-based rather than ECC-based, the device-side computation per session is small; the trade-off is that its security strength depends on the hash-centric design and the access-control structure of the cluster^36^. proposed a two-factor ECC-based protocol incorporating hash functions and timestamps for mutual authentication and replay resistance. While efficient for individual sessions, their scheme does not accommodate clustered or batch-based authentication flows^37^. offered LA-IoMT, which employs lightweight hash chaining to simplify verification, but its static per-device structure lacks scalability in real-time clustered hospital settings.

### Mobility/privacy-focused variants

Reference^20^ introduced the LEMAP protocol, a location-aware lightweight scheme optimized for mobile IoMT devices. It achieves lower latency through spatial metadata and one-time ECC operations, yet offers no support for critical/non-critical device categorization or batch-level authentication, limiting its suitability for latency-sensitive, high-concurrency clinical deployments^13^. developed an ECC-centric protocol using pseudonymous identities and ephemeral secrets. While this enhances privacy (e.g., identity protection) by using pseudonymous identities and ephemeral secrets, it still treats each device independently and does not support authentication request aggregation or session key reuse within a group context.

Recent IoMT authentication and session key generation protocols increasingly combine privacy goals (e.g., untraceability) and stronger adversary modeling with efficiency constraints; comprehensive taxonomies and critical reviews are available^18^, and several recent schemes target telecare medical systems and medical consortium settings^38,39^. We position SELAM as a complementary design point focused on selective-ECC for critical-device authentication, emphasizing minimal online cost rather than blockchain support or full untraceability.

Synthesis across these threads yields three persistent limitations. First, even though ECC is lighter than RSA, invoking ECC across registration, login, and mutual-authentication phases inflates device-side compute and end-to-end delay per session; under concurrency, this becomes the dominant bottleneck^14,22,29,35^. Second, scalability techniques such as group selectivity, batch signatures, and fog or blockchain mostly lower the cost on the verifier, the network, or the overall system, and they simplify auditing, but they usually leave the cryptographic work on the device during the session unchanged^16,31–33^. Third, lightweight primitives (hash/HMAC, XOR, timestamp freshness) are often treated as auxiliaries rather than full substitutes for ECC in non-critical phases, leaving avoidable overhead on constrained nodes^19,35^. Methodologically, several works emphasize message/operation counts or system throughput (particularly in blockchain/fog settings) without publishing per-phase, per-device timings on constrained hardware, which obscures the true cost driver for battery-limited medical sensors. A representative recent scheme is ALMASH^14^, which partitions authentication into five phases using ECC, hashed tokens, and Shamir’s Secret Sharing. It achieves anonymity and lightweight operation, but its key limitations lie in (i) treating each device independently, (ii) not incorporating aggregation or batch operations, and (iii) lacking criticality-aware key distribution.

From the comparison in Table 1, it is evident that while several schemes utilize lightweight cryptographic primitives such as hashing, ECC, or PUFs, none fully address the joint requirements of scalable multi-device authentication, group-based session key derivation, and real-time differentiation between critical and non-critical medical data streams. Notably, although^14^ achieves a balanced lightweight footprint through selective ECC and Shamir’s Secret Sharing, it still handles each device independently and lacks batch authentication support. Similarly, schemes by^13,36,37^ focus on per-session mutual authentication but do not implement clustered key reuse or asynchronous aggregation. Park and Park’s early context-aware group authentication concept lacks formal cryptographic integration and remains unsuitable for healthcare-grade deployments^30^. These findings validate the necessity of a novel authentication framework that integrates selective ECC at critical phases, lightweight hash and XOR operations during runtime, and a cluster-aware session key derivation process that enhances scalability, latency resilience, and energy efficiency in constrained IoMT environments.

In contrast to ECC-phase-heavy schemes, SELAM relocates ECC to one-time registration (user and device) and executes only lightweight operations such as hash/HMAC, XOR, and timestamp freshness checks on the online path (login and mutual authentication/session establishment). This selective ECC placement preserves the standard security goals evaluated in our threat model (mutual authentication; resistance to replay, impersonation, and MitM considered in the experiments) while explicitly eliminating public-key work from the critical path, where prior art pays most of its latency and energy. Conceptually, SELAM aligns with the accepted advantages of ECC over RSA^11^ but diverges from mainstream designs by changing where ECC occurs in the pipeline rather than adding further ECC-based online phases.

**Fig. 1:**
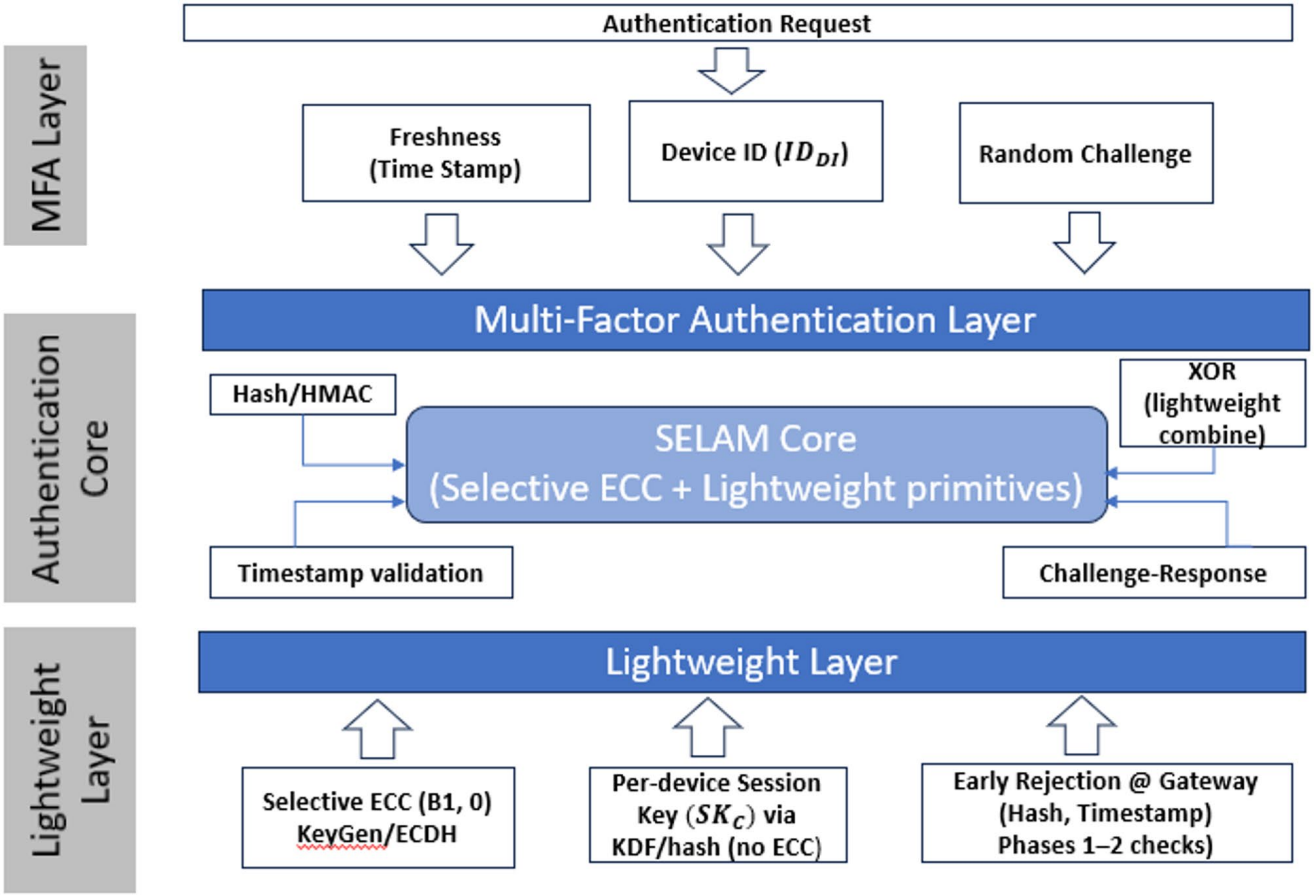
SELAM system model. ECC is confined to one-time registration (Phases B1 and 0). All online phases use lightweight primitives (Hash/HMAC, XOR, timestamp freshness) with early rejection at the gateway. Session keys ($\:{\boldsymbol{S}\boldsymbol{K}}_{\boldsymbol{C}})$ are derived per device via KDF/hash without per-message ECC.

The security implications of these design choices are mapped phase-by-phase in Section VI and summarized in Table 9, while the performance impact is evaluated empirically in Section IV (Fig. 1).

## System design

### Protocol model

**Fig. 2:**
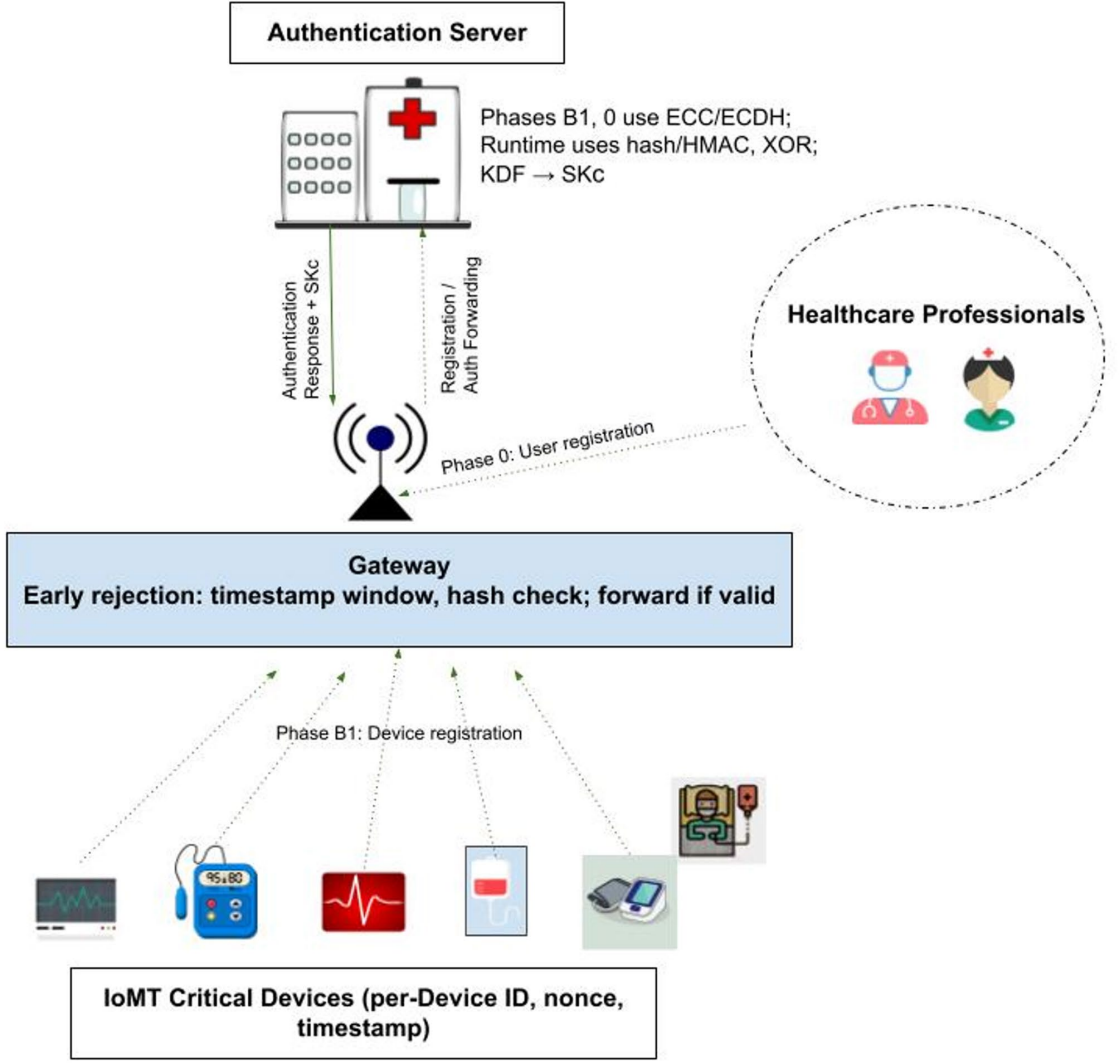
SELAM implementation.

Figure 2 presents the Selective Elliptic Curve Lightweight Authentication Model, SELAM, for the Internet of Medical Things. The design preserves the cryptographic strength of elliptic curve methods while reducing online cost by confining all elliptic curve operations to registration. Runtime authentication relies only on lightweight primitives. This choice directly addresses latency and energy constraints in clinical environments where medical devices are resource-constrained, and responses must be delivered in real time.

The model is organized conceptually into three layers. (i) The Multi Factor Authentication layer accepts user credentials that include identity $\:\left({ID}_{U}\right)$, password $\:\left({PW}_{U}\right)$, and biometric $\:\left({B}_{U}\right)$, together with a timestamp $\:\left(T\right)$ and the identity of the critical medical device, denoted $\:{ID}_{{D}_{i}}$. During registration, these inputs are bound to long-term state through elliptic curve key generation, so that only genuine users and devices are enrolled. (ii) The Authentication Core executes during normal sessions in Phases 1 to 6 and uses computationally inexpensive operations. Hash or HMAC provides integrity, XOR serves as a lightweight combiner where needed, and timestamps enforce freshness. A challenge and response exchange confirms liveness and prevents replay. When verification succeeds, the parties establish a secure channel using a session key $\:{K}_{\mathrm{sess}}\left({D}_{i}\right)$, and no elliptic curve computation is performed in these online phases. (iii) The Lightweight Security layer restricts elliptic curve work to Phase B1 for device registration and Phase 0 for user registration, where long-term secrets with the Authentication Server are created through ECDH. Session keys are refreshed across sessions via KDF/HMAC-based updates from $\:{K}_{\mathrm{seed}}\left({D}_{i}\right)$without invoking further ECC. The gateway performs basic freshness and syntactic checks before relaying to the server.

Four entities participate in the protocol, namely the user, the critical medical device, the gateway, and the authentication server. The adversary is assumed to control the communication channel and can intercept, modify, or replay messages. Under the hardness of the elliptic curve discrete logarithm problem and the one-way property of the hash function, SELAM provides confidentiality, mutual authentication, and resistance to impersonation and replay.

This work evaluates SELAM under per-device authentication with $\:{K}_{\mathrm{sess}}\left({D}_{i}\right)$ derived from the ECDH result established at registration. The system view in Fig. 2 follows this scope. Critical medical devices, such as electrocardiogram and blood pressure monitors, transmit authentication requests that carry $\:{ID}_{{D}_{i}}$, a nonce-based challenge, and a timestamp to the gateway. The gateway verifies freshness and integrity with lightweight checks and forwards only valid requests. The authentication server maintains registration state, performs elliptic curve operations only in Phases B1 and 0, and manages session key establishment for runtime phases. Healthcare professionals then access services over sessions protected by $\:{K}_{\mathrm{sess}}\left({D}_{i}\right)$ after a successful challenge and response. By arranging the workflow in this manner, SELAM confines computationally intensive elliptic curve processing to registration while achieving recurring authentication through lightweight primitives, thereby delivering a secure and efficient protocol suitable for the Internet of Medical Things. Table 1 summarizes representative IoMT authentication schemes (including ALMASH and 3ECAP) along the design axes most relevant to ECC runtime cost and deployment features.

The proposed algorithm of SELAM mechanisms is explained in detail as follows.

### Threat model and assumptions

We adopt a Dolev–Yao network adversary model, in which the attacker has full control over the communication channel, including the ability to eavesdrop, intercept, replay, delay, modify, and inject messages. The adversary is computationally bounded and cannot break standard cryptographic primitives (e.g., hash functions, MACs, ECC). The authentication server is assumed to be trusted, and long-term secrets are securely stored unless explicitly stated otherwise. Replay and impersonation attacks are explicitly evaluated under bounded clock skew (Δ) and nonce freshness assumptions, consistent with common IoMT threat models. This adversary model follows standard Dolev–Yao assumptions and aligns with recent IoMT threat classifications^40^ adopted in medical IoT security studies.

Corruption and trust assumptions. The AS is trusted and maintains long-term credential bindings established during registration. The GW enforces basic freshness checks (timestamp window Δ and nonce/replay cache) and is not compromised in the evaluated setting. Physical capture and side-channel leakage (power/EM/timing) are out of scope; however, the design minimizes per-session secret exposure by restricting ECC to one-time registration and using lightweight online computations.

Cryptographic assumptions. The hardness of the elliptic-curve discrete logarithm problem (ECDLP) holds on the selected curve; the hash/HMAC construction is modeled as a secure one-way and collision-resistant primitive; and the KDF behaves as a pseudorandom function over its inputs (seeds, nonces, and timestamps).

Attacks considered in the evaluation include replay and impersonation, as well as man-in-the-middle attempts limited to message interception, modification, and replay within the Dolev–Yao network model (e.g., reuse of previously observed messages or nonces). Denial-of-service effects are evaluated at the protocol level by injecting elevated authentication request rates that stress freshness checks, replay caches, and bounded retry mechanisms; volumetric or network-layer denial-of-service attacks are outside the scope of this work. All parameters, including the freshness window Δ, nonce length, and attack rates, are disclosed in the experimental setup.

### Development of SELAM system

This section introduces a lightweight authentication framework designed for the Internet of Medical Things. The design reduces computational complexity, minimizes communication cost, and supports real-time scalability for critical medical devices. SELAM preserves the cryptographic strength of elliptic curve methods while reducing online cost by confining elliptic curve operations to registration. All runtime interactions use lightweight primitives.

The proposed framework builds upon the phased model used in ALMASH^14^ but introduces key enhancements: (i) selective usage of Elliptic Curve Cryptography (ECC) only during registration phases (device and user), (ii) reliance on lightweight cryptographic operations such as hashing, XOR, and timestamp validation for runtime authentication to minimize repeated overhead, and (iii) streamlined message flows that remove redundant cryptographic steps without weakening security guarantees. Unlike existing ECC-based IoMT authentication schemes that repeatedly invoke ECC across multiple phases, SELAM confines ECC strictly to initial registration while using lightweight primitives during all subsequent login and authentication operations. This design significantly reduces computational and communication costs, supporting real-time deployment in hospital IoMT settings where latency and resource efficiency are critical.

Although the general SELAM framework supports cluster-based aggregation, in this paper, we restrict to the single-device instantiation: the Cluster Head (CH) role degenerates to an individual device $\:{D}_{i}$, and no group-level ECC is performed. ECC operations appear only in Phases B1 and 0, while Phases 1–6 rely on lightweight primitives. Group-level extensions will be made in future work. The entire authentication framework is divided into the following key phases:

**Table 1:** Comparative summary of representative authentication schemes and SELAM.

Scheme	System model/setting	Auth factors (as stated)	Group/cluster/batch	Online asymmetric cryptography? (scope)	Online primitives	Online message exchanges (rounds)	Security + verification
ALMASH^14^	IoHT/IoMT user–things authentication via registration gateway and healthcare server; ECC + Shamir secret sharing for scalable multi‑device access.	Password-based user auth + device secrets (no biometrics stated)	Scalability via secret sharing; supports authenticating multiple things	Yes (ECC point operations during authentication; plus symmetric protection)	ECCmul; Hash; SymEnc/Dec; XOR; SSS	Not explicitly summarized (multi-entity: HT↔RG↔HS and U↔RG)	MA; ANON(UTA); FS; DOS; IA; MITM; OPA; FSV(AVISPA)
Cross-hospital ECC on ledger^32^	Cross-hospital doctor/patient authentication with blockchain + smart contracts; supports decentralization and cross‑cluster applicability.	n‑factor	Cross‑cluster; decentralized deployment (ledger)	Yes (ECC + blockchain operations online)	ECCmul; Hash; SymEnc/Dec; FE; BCtx/SC	AM1–AM4 (4 msgs)	3 F (three-factor secrecy); OGA; IA; RA; MITM; SCL; DNC; SVA; DESYNC; MA; SKS; KSKA; PFS; ANON(doc/pat); UNLK; DECENT; SCALE; CROSS; FSV(RoR/ROM)
ESEAP^22^	IoT/WSN lightweight mutual authentication using enhanced EC‑ElGamal (ECEG) style operations; emphasis on efficient encoding/parameter exchange.	Two‑party mutual authentication	No	Yes (ECC‑ElGamal operations online)	ECCenc/dec (ECEG); ECCmul; Hash	1 RTT (2 msgs)	MA; CONF; INT; FSV(Scyther)
Fog-enabled blockchain auth.^33^	Fog-enabled IoT authentication with blockchain support; evaluated with NS‑3; explicit security feature comparison across 16 features (SF1–SF16).	Credential + PUF + FE elements (multi-factor components used)	No (per-user/per-session); fog + cloud infrastructure	Yes (ECC scalar multiplication/addition online)	Hash; FE; PUF; ECCmul; ECCadd; SymEnc/Dec; RNG; CM; BC(ledger)	4 msgs	IA; INS; OGA; SCL; RA; MITM; DOS; MA + SKS; UTR; DESYNC; PINS; FS; SS‑RNL; SVA; ANON; KCI; FSV(eCK‑style + table SF1–SF16)
Four-factor ECC + PUF^29^	IoT/IoMT four-factor authentication leveraging ECC + PUF + biometrics + password/smart‑card; formal BAN + ProVerif validation reported.	4 F (PW + smart card/device + biometrics + PUF)	No	Yes (ECC operations in mutual authentication)	ECCenc/dec or ECCmul; Hash/Bio‑hash; FE; PUF; RNG	M3–M6 (4 msgs)	MA; SKA; FS(fwd + bwd); ANON; UTR; BTS; SCL; 4 F; FSV(BAN+ProVerif)
Group over blockchain + fog^31^	IoMT group authentication using multi‑level blockchain and fog; smart contracts (global/local chains); evaluated with AVISPA; aims at scalable group verification.	Not emphasized	Yes (group authentication; multi-level blockchain)	Yes (ECC-based lightweight enc/dec mentioned for devices)	ECCenc/dec; Hash; BCtx/SC; TS	Variable (transaction-based; not directly stated)	SCALE; LWT; DIM; TE; SDC; FSV(AVISPA)
ID-LVEAS^16^	IoMT locally verifiable batch authentication via ID-based locally verifiable aggregated signcryption; provides privacy & integrity with provable security.	Not multi-factor; signcryption-based	Yes (batch + local verification)	Yes (pairing-based aggregated signcryption/signatures)	Pair; Exp; Hash; Sign/Verify; AggSign/AggUnsigncrypt	N/A (non-interactive batch verification)	PRIV; INT; EU‑CMA; LR; FSV(q‑BDHI/ROM)
Selective group auth.^30^	Selective group authentication for IoT-based medical information systems (group aggregator selects/verifies members).	Not explicitly multi-factor (smart-card style setting)	Yes (selective group)	No (lightweight, symmetric/hash style)	Hash; XOR; TS	Variable	IA; MITM; OPA; (no MA/ANON/FS/DOS in ALMASH comparison); FSV(–)
3ECAP (cluster, three-factor)^35^	Cluster-based IoMT authentication with access-list clustering; smart card + gateway; formal verification reported with ProVerif.	3Factor	Yes (cluster-based access control)	No online ECC in reported cost table (hash + FE dominated)	Hash; FE; RNG	M1–M3 (3 msgs)	IA(U/G/S); SVA; RA; DOS; SCL; ANON; UTR; FS; MA; SKA; FGAC; FSV(ProVerif)
SELAM (this work)	Selective‑ECC lightweight authentication for critical IoMT devices: ECC confined to registration; runtime avoids online asymmetric ops; dataset-driven simulation.	Credential + device secret (selective-ECC design)	No (per-device authentication)	No (selective-ECC: registration-only ECC)	Hash; XOR; TS; (optional HMAC in validation)	3 RTT (≈ 6 msgs)	MA; SKS; RA; IA; MITM; OGA

Notes: RTT = round trip time; msgs = one-way protocol messages. Where an article does not provide an explicit message sequence summary, the value is marked as variable/not stated.

**Legend (Security)**: MA=Mutual Authentication; ANON=Anonymity; UTA=User & Things Anonymity; UTR=Untraceability; UNLK=Unlinkability; FS/PFS=Forward/Perfect Forward Secrecy; SKS=Session Key Secrecy; SKA=Session Key Agreement; OGA=Offline Guessing Attack resistance; OPA=Online/Offline Password Attack resistance; IA=Impersonation Attack resistance; MITM = Man-in-the-Middle resistance; DOS = DoS resistance; INS=Insider attack resistance; PINS=Privileged insider resistance; SCL=Stolen smart card/device resistance; DNC=Device node capture resistance; SVA=Stolen verifier resistance; DESYNC=Desynchronization resistance; KSKA=Known session key attack resistance; SS‑RNL=Session-specific random number leakage resistance; BTS=Biometric template security; FGAC=Fine-grained access control; DIM=Decentralized identity management; TE=Time efficiency; SDC=Single-device compromise isolation; DECENT=Decentralization; SCALE=Scalability; CROSS=Cross-cluster applicability; LR=Leakage resilience; FSV=Formal security verification/proof; ROM=Random Oracle Model; RoR=Real-or-Random; EU‑CMA=Existential Unforgeability under Chosen-Message Attack.

**Legend (Primitives)**: ECCmul = ECC point/scalar multiplication; ECCadd = ECC point addition; ECCenc/dec = ECC-based encryption/decryption; ECEG = EC‑ElGamal; Hash=cryptographic hash; XOR=exclusive‑OR; HMAC=keyed hash; FE=fuzzy extractor; PUF=physical unclonable function; SymEnc/Dec=symmetric encryption/decryption; RNG=random number generation; CM=chaotic mapping; Pair=bilinear pairing; Exp=group exponentiation; Sign/Verify=digital signature operations; AggSign/AggUnsigncrypt=aggregate (sign)cryption operations; BCtx/SC=blockchain transaction/smart contract call; SSS=Shamir secret sharing; TS=timestamp/freshness check.

i. Phase A – Initial setup and parameter configuration.
ii. Phase B1 – Device registration (with ECC key generation).
iii. Phase B2 – Cluster setup and Group-ID assignment (not instantiated in this evaluation).
iv. Phase 0 – User registration (with ECC).
v. Phase 1 – User login.
vi. Phase 2 – Authentication initiation at gateway (early rejection: timestamp window, hash/HMAC).
vii. Phase 3 – Device authentication request submission.
viii. Phase 4 – Per-device session key derivation and distribution (KDF/HMAC, no ECC).
ix. Phase 5 – Mutual challenge-response validation (hash/XOR based).
x. Phase 6 – Secure session establishment and key distribution.

Cryptographic symbols and operations used throughout SELAM are defined in Table 2, following conventions common to ECC-based authentication in IoT/IoMT^14,36^.

**Table 2:** Proposed SELAM cryptographic symbols and description.

Symbols	Description
A. Entities and ECC keys	
$\:{ID}_{U},{ID}_{{D}_{i}}$	Identities
$\:{U,D}_{i},GW,AS$	User, device, gateway node, authentication server
$\:{d}_{u},{d}_{{D}_{i}}$,$\:{d}_{GW},\:{d}_{AS}$	ECC private keys of user, device, gateway, authentication server
$\:{P}_{u},{P}_{{D}_{i}}$,$\:{P}_{GW},\:{P}_{AS}$	ECC public keys of user, device, gateway, authentication server
$\:{K}_{GW-AS}$	Symmetric GW–AS channel key (derived once at registration via ECDH; used to authenticate registration acks)
B. Freshness and cryptographic primitives	
$\:{N}_{U}$,$\:{N}_{GW}$,$\:{N}_{{D}_{i}}$	Random nonces generated by user, gateway, or device
$\:{\:T}_{j}$	Timestamps in phase$\:j$(freshness window Δ)
$\:h(\cdot )$	Cryptographic hash function
$\:\oplus\:$	XOR operation used in token generation
||	Concatenation
HMAC	Keyed hash (Symmetric MAC)
KDF	Key-Derivation Function for deriving new keys
C. Long-term/session material	
$\:{K}_{seed}{(D}_{i})$	ECC-derived long-term seed key from registration
$\:{K}_{sees}{(D}_{i})$	Per-device session key
$\:{ST}_{U}$	Session token generated after user login
$\:\tau\:$	HMAC tag on online messages

These notations will be used consistently in the following phase-by-phase description of the proposed authentication scheme. For clarity, the algorithms are presented in their general form, including cluster-head notation. In this paper’s evaluation, $\:CH\:=\:{D}_{i}$ (single-device case), and Phase 4 is implemented using a KDF instead of group ECC.

**Figure:**
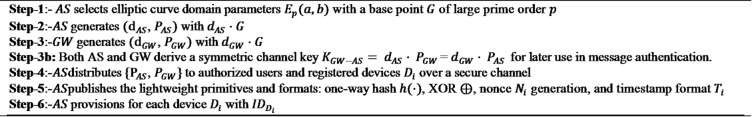
**Algorithm-1.1**: System-parameters announcement message (AS → GW, $\:{D}_{i}$) - *(Phase A)*.

The initial setup phase establishes the fundamental parameters required for the secure operation of the proposed SELAM system as described in Algorithm-1.1. The process begins by selecting a secure elliptic curve $\:{E}_{p}(a,b)$ over a large finite field, with a generator point $\:G$ of prime order $\:p$. This choice ensures robustness against elliptic curve discrete logarithm attacks, forming the cryptographic backbone of the system.

Both the Authentication Server (AS) and the Gateway (GW) independently generate ECC key pairs $\:{\mathrm{(d}}_{AS}\mathrm{,\:}{P}_{AS})$ and $\:{\mathrm{(d}}_{GW}\mathrm{,\:}{P}_{GW})$ These keys are critical for performing selective ECC operations during registration and group key generation phases, while being excluded from frequent online authentication to reduce computational load.

Once generated, the public keys $\:{\mathrm{P}}_{AS}\:$and $\:{P}_{GW}$ are distributed securely to authorized users and registered medical devices. This enables mutual trust and ensures that subsequent authentication exchanges can be securely verified. In parallel, lightweight primitives are also standardized: hash functions for timestamp verification, XOR for session token confirmation, and nonce generation for replay protection.

Finally, each critical medical device $\:{D}_{i}$ is provisioned with its unique identifier with $\:{ID}_{{D}_{i}}\:$allowing devices to be mapped to specific clusters. This preparatory phase guarantees that when the authentication workflow begins, all entities possess the necessary cryptographic parameters and lightweight tools for selective ECC-based authentication.

In the Algorithm-1.2, the device registration phase, each critical medical device $\:{D}_{i}\:$establishes its long-term cryptographic identity within the system. The device generates an ECC key pair ($\:{d}_{d}$,$\:{P}_{d}={d}_{D}\cdot \:G$) and forms the registration payload $\:{M}_{1}$=($\:{ID}_{{D}_{i}}$,$\:{N}_{i}$,$\:{T}_{i}$) with integrity tag $\:{H}_{1}$=$\:h$($\:{M}_{1}$). The tuple {$\:{M}_{1,\:}{H}_{1},{P}_{D}\:\}$ is encrypted under the AS public key and sent via the gateway. The gateway performs lightweight checks (freshness of $\:{T}_{i}$, basic format/duplicate $\:{ID}_{{D}_{i}}$) and forwards the ciphertext unchanged to the AS. At the AS, a single ECC operation is performed to derive the device’s seed key $\:{K}_{seed}$=$\:h{(d}_{AS}\cdot\:{P}_{D}\parallel\:{ID}_{{D}_{i}}\parallel\:{T}_{i}).\:$ The AS returns and an acknowledgement $\:{M}_{2\:}=\left\{{T}_{2},\tau\:\right\},\:\:$upon receipt, $\:{D}_{i}$ verifies $\:\tau\:$ and stores only $\:{K}_{seed}\left({D}_{i}\right)$as its long-term secret. All subsequent authentications avoid online ECC: Phases 1,3,5, and 6 rely on lightweight primitives (hash/HMAC, XOR, timestamps), and per-session keys are derived from $\:{K}_{seed}\left({D}_{i}\right)$via KDF. This selective ECC design minimizes computational cost while preserving mutual authentication.

**Figure:**
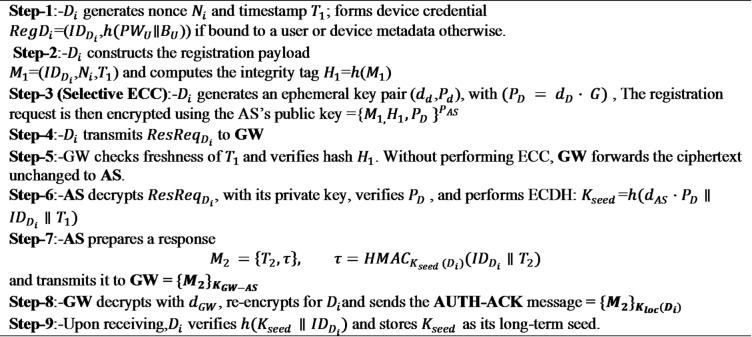
**Algorithm-1.2** Device Registration Message ($\:{D}_{i}$ → GW→AS) — *(Phase B1*,* ECC)*.

In this work we evaluate the per-device instantiation; Algorithm-1.3 (cluster setup) is present for continuity but not exercised. We interpret clusters as singletons (|$\:{C}_{h}|=1)$, set $\:{GID}_{h}:=\perp\:$and identify $\:CH\:\equiv\:\:GW$. No GW–CH channel key is established. All authentication in later phases is per-device: keys derive from $\:{K}_{seed}\:\left({D}_{i}\right)$ and Phase-4 yields $\:{K}_{seed}\:\left({D}_{i}\right)$ (no group keying).

**Figure:**
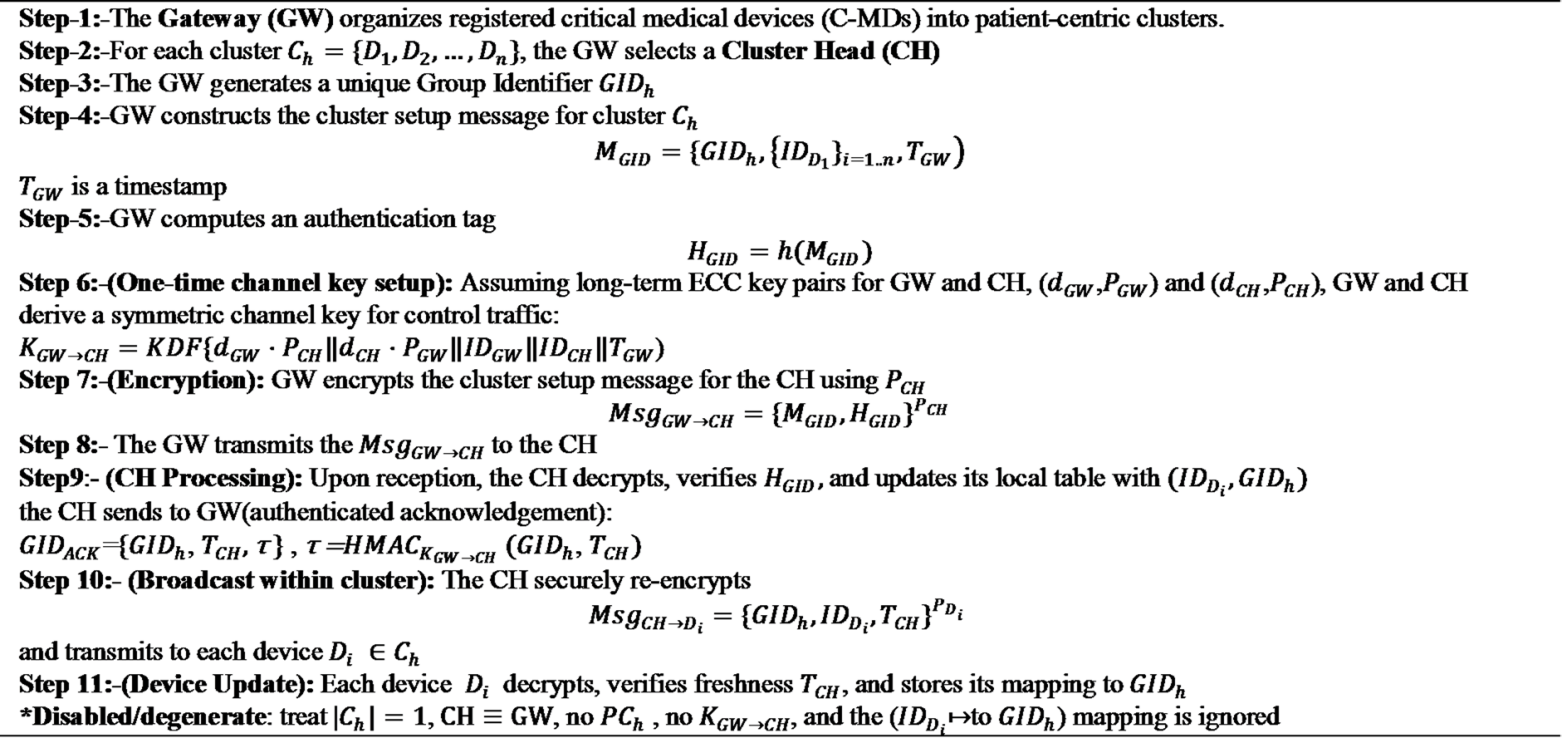
**Algorithm-1.3** Cluster-Setup and GID Assignment Message (GW → $\:{\boldsymbol{D}}_{\boldsymbol{i}}$)-*(Phase B2)*.

In Algorithm-1.4, the user registration phase establishes a secure and multifactor binding between a healthcare professional and the authentication infrastructure. The combination of password $\:{PW}_{U}$, and biometric$\:{\:B}_{U}$ ensures that the hashed identity $\:{H}_{U}$ is resistant to replay and impersonation attacks. By generating an ECC key pair $\:({d}_{U},{P}_{U})$, the user anchors their identity to a cryptographic credential, which is validated during subsequent login attempts.

The gateway operates as a lightweight verifier and trusted intermediary: it checks message integrity and freshness, then forwards the verified registration message $\:{M}_{0}=\left({ID}_{U},{H}_{U},{P}_{U},{T}_{U}\right)\:$to the authentication server (AS) with an authentication tag $\:\tau\:={HMAC}_{{K}_{GW-AS}}\left({M}_{0}\right)$. At the AS, a single verification step authenticates the registration over the protected GW–AS channel; the AS stores $\:{(ID}_{U},{H}_{U},{P}_{U})$ for future sessions (no plaintext v or $\:{B}_{U}\:$retained). This selective-ECC design confines public-key cost to registration; all subsequent login/authentication phases rely only on low-cost hash/XOR primitives, meeting this work efficiency goals.

**Figure:**
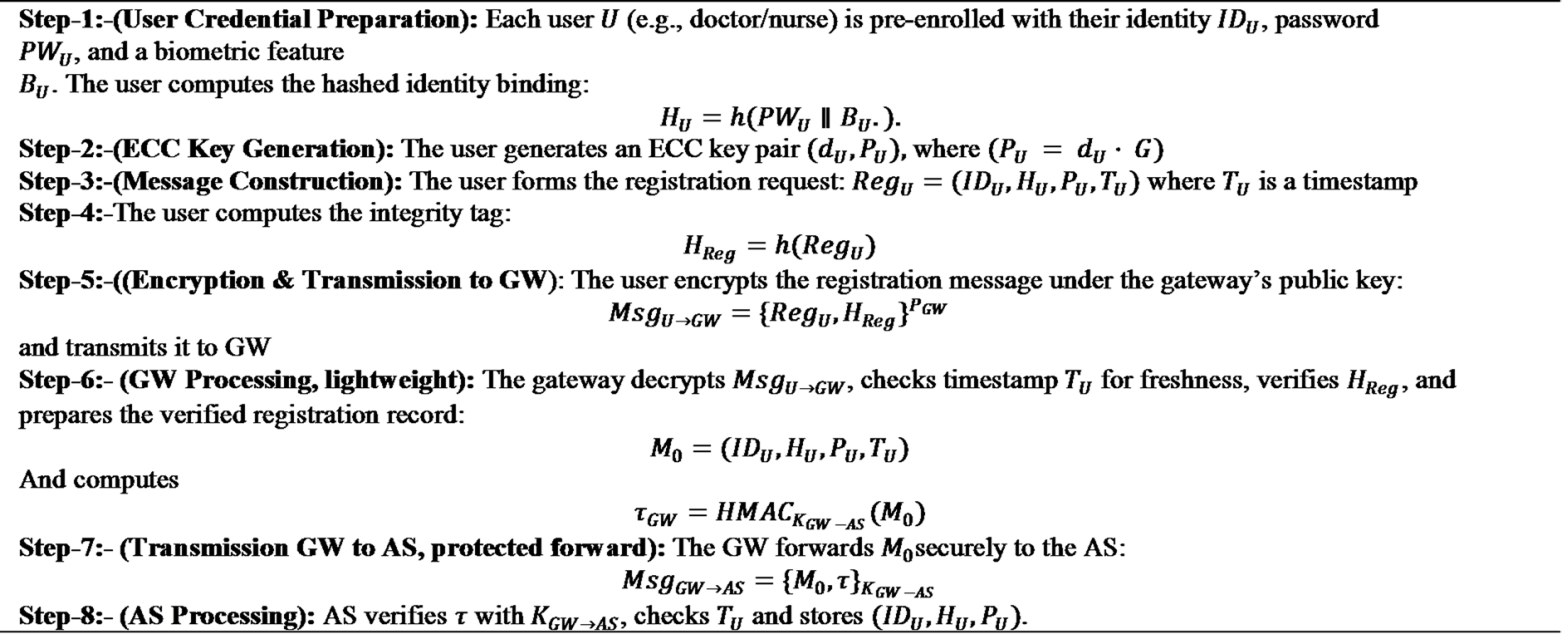
**Algorithm-1.4** User-registration message (U→GW→AS)-Phase 0 (with ECC).

Algorithm-1.5 authenticates the user with only lightweight primitives. The client computes the multifactor $\:{H}_{U}=h\left({PW}_{U}\parallel\:{B}_{U}\text{}.\right),$ adds nonce/timestamp for freshness and sends an AUTH-LOGIN message to the gateway. The gateway performs hash and timestamp checks (no ECC), then forwards the verified login record to the AS using symmetric protection (HMAC) under the GW–AS channel key $\:{K}_{GW-AS}$ that was derived once during Phase A by a single ECDH. The AS verifies the tag and issues a session token $\:S{T}_{U}$=$\:KDF({H}_{U}\parallel\:{N}_{U}\parallel\:{T}_{3})$.This token will be used in subsequent phases to support efficient, lightweight mutual authentication with devices.

This design adheres to the selective ECC principle: no new ECC operations are introduced during login. ECC is confined to initial registration, while routine login leverages hash, XOR, and HMAC operations, keeping computational overhead low.

**Figure:**
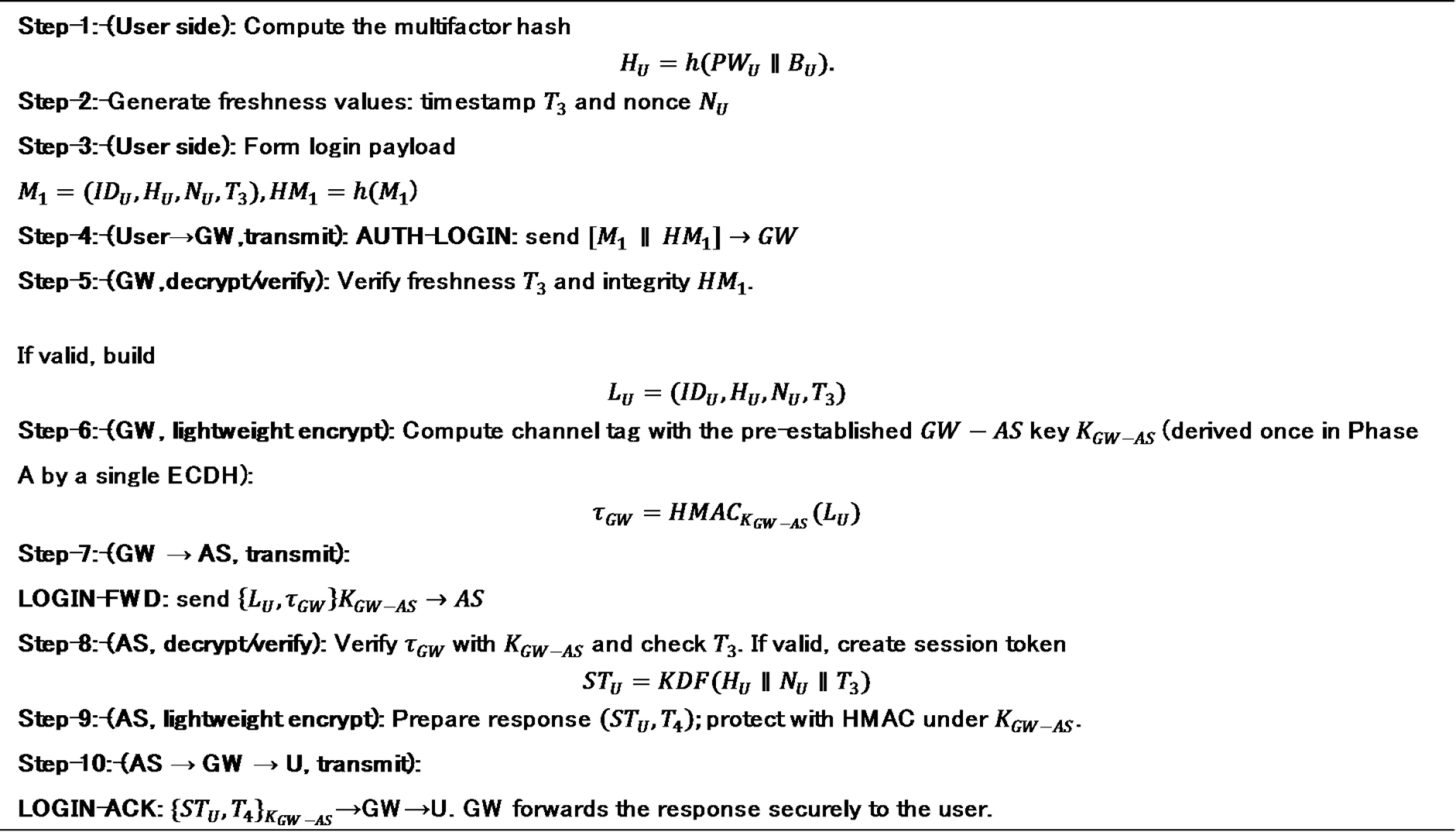
**Algorithm-1.5** User login message (U → GW → AS)– phase 1 (lightweight only; no ECC).

Algorithm-1.6 defines the AUTH-REQ message exchange, which allows an authenticated user to trigger a per-device authentication round. Once the user has successfully logged in (Phase 1), they generate a fresh nonce and timestamp and combine them with their session token $\:S{T}_{U}$, and submit the AUTH-REQ message to the gateway. The gateway verifies integrity and freshness, then forwards the validated request to Phase 3 (device challenge) — no $\:GW\to\:CH$ hop, no $\:{K}_{GW-CH}$. Optionally, GW records an audit tag $\:{\tau\:}_{GW}$ No ECC is introduced online.

**Figure:**
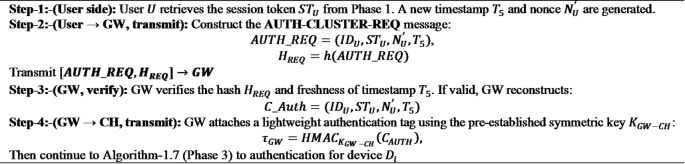
**Algorithm-1.6** AUTH_REQ Message (U → GW )-Phase 2.

Algorithm-1.7 details the AUTH_DEVICE_REQ exchange. After Phase-2, the gateway (standing in for CH) challenges device $\:{D}_{i}$ using a lightweight, authenticated request that includes the user’s token $\:{ST}_{U}$, a gateway nonce $\:{N}_{GW}$, and a timestamp $\:{T}_{6}$. The device verifies freshness, responds with a hash over ($\:{ST}_{U}{,N}_{GW}\:,{N}_{{D}_{i}},{T}_{6})\:$and both sides confirm membership by recomputing the response. All operations use hash/HMAC and a symmetric key derived from the device’s registration seed; no ECC appears online.

**Figure:**
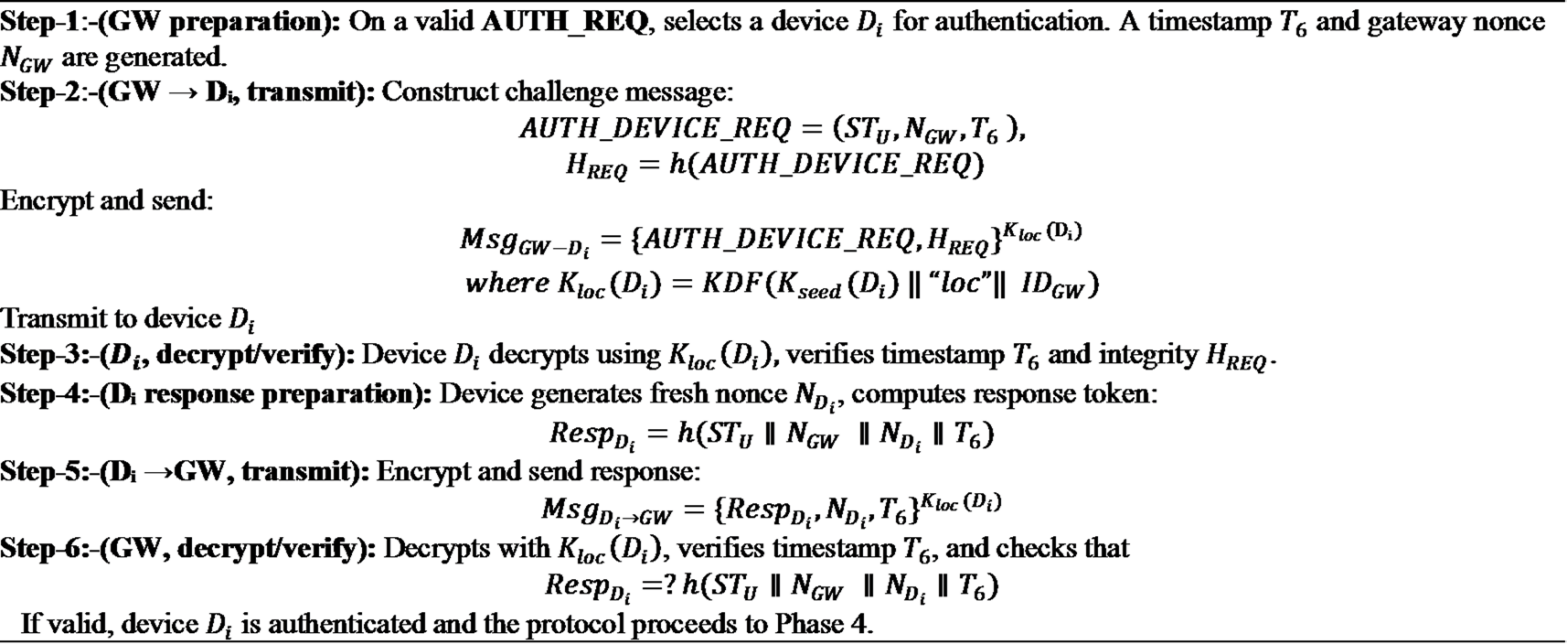
**Algorithm-1.7** AUTH_DEVICE_REQ message (GW ↔ Device D_i_)-phase 3 (lightweight only).

For algorithm-1.8, in this phase 4, the gateway establishes a fresh session key with the device $\:{D}_{i}$ to secure subsequent authentication and data exchange. Unlike group-based scenarios that require cluster-level ECC (addressed in later work), SELAM’s instantiation performs no online ECC operations. Instead, both the gateway and device derive the same session key $\:{K}_{sess}$ using a deterministic key derivation function (KDF) seeded with the device’s long-term keying material established in Phase B1 (the gateway stores $\:{K}_{seed}\left({D)}_{i}\text{}\right);\:$user registration (Phase 0) is orthogonal to Phase-4.

The gateway initiates the process by transmitting a session-key request message protected with an HMAC over its long-term seed. Upon receipt, the device validates the HMAC, checks the freshness of the included timestamp, and if valid it derives $\:{K}_{sess}$ locally using the same KDF inputs. The device then acknowledges the key agreement by returning an HMAC-protected confirmation message. Finally, the gateway verifies this acknowledgment, completing synchronization of $\:{K}_{sess}$.

Through this design, both entities establish a common ephemeral session key without requiring per-device ECC operations during runtime. This preserves confidentiality, integrity, and mutual key confirmation while adhering to SELAM’s principle of confining ECC to registration. The derived session $\:{K}_{sess}\:$is subsequently used in Phases 5 and 6 for mutual challenge–response validation and secure session establishment.

**Figure:**
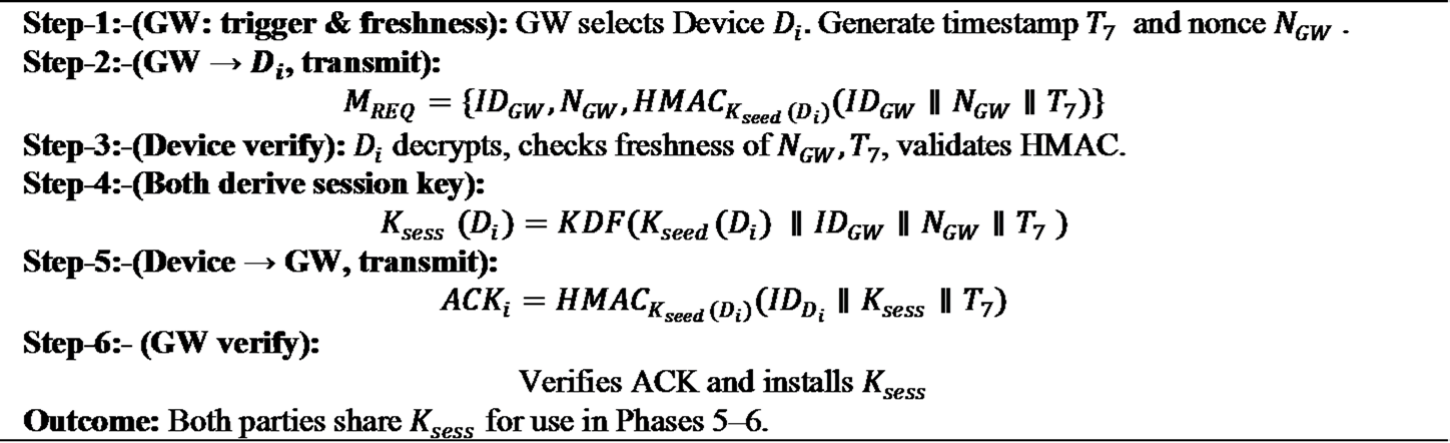
**Algorithm-1.8** Session key derivation and distribution (GW ↔ Device $\:{D}_{i}$) – Phase 4.

In Algorithm-1.9, this phase provides per-device mutual authentication by proving that both the gateway and device $\:{D}_{i}.\:$ possess the session key $\:{K}_{sess}\left({D}_{i}\right)$ established in Algorithm-1.8. The gateway generates a fresh challenge $\:{M}_{CH}=\left({N}_{GW}^{{\prime\:}}\text{},{T}_{11}\:\right)\:$and attaches a keyed integrity tag $\:{H}_{CH}={HMAC}_{{K}_{sess}\left({D}_{i}\right)}{(M}_{CH})$. The challenge is sent to $\:{D}_{i}\:$ over the symmetric transport key $\:{K}_{loc}\left({D}_{i}\right)$(derived from the device’s registration seed).Upon receipt, $\:{D}_{i}$ decrypts, checks freshness of $\:{T}_{11}$, and verifies $\:{H}_{CH}$. It then samples its own fresh nonce $\:{N}_{{D}_{i}}^{{\prime\:}}$and computes the response $\:{Resp}_{\:{D}_{i}}={HMAC}_{{K}_{sess}\left({D}_{i}\right)}({N}_{GW}^{{\prime\:}}\oplus\:{N}_{{D}_{i}}^{{\prime\:}}\parallel\:{T}_{11})$, which binds both nonces and the timestamp, ensuring liveness and replay resistance. The device returns $\:{(Resp}_{\:{D}_{i}},{N}_{{D}_{i}}^{{\prime\:}},{T}_{11})\:$to the gateway, protected under $\:{K}_{loc}\left({D}_{i}\right)$. The gateway decrypts, recomputes $\:{Resp}_{{D}_{i}}^{{\prime\:}}$ the same way and accepts only if $\:{Resp}_{{D}_{i}}^{{\prime\:}}={Resp}_{\:{D}_{i}}$. To provide explicit key confirmation, the gateway finishes by sending a confirmation token $\:{Conf}_{CH}={HMAC}_{{K}_{sess}\left({D}_{i}\right)}({ID}_{{D}_{i}}\parallel\:{N}_{GW}^{{\prime\:}}\parallel\:{N}_{{D}_{i}}^{{\prime\:}}\parallel\:{T}_{11})$ which the device verifies before concluding the phase. The entire exchange uses hash/HMAC and symmetric encryption only; no ECC operations occur online. The design achieves freshness, liveness, and mutual possession of $\:{K}_{sess}({D}_{i}$) with minimal computational cost.

**Figure:**
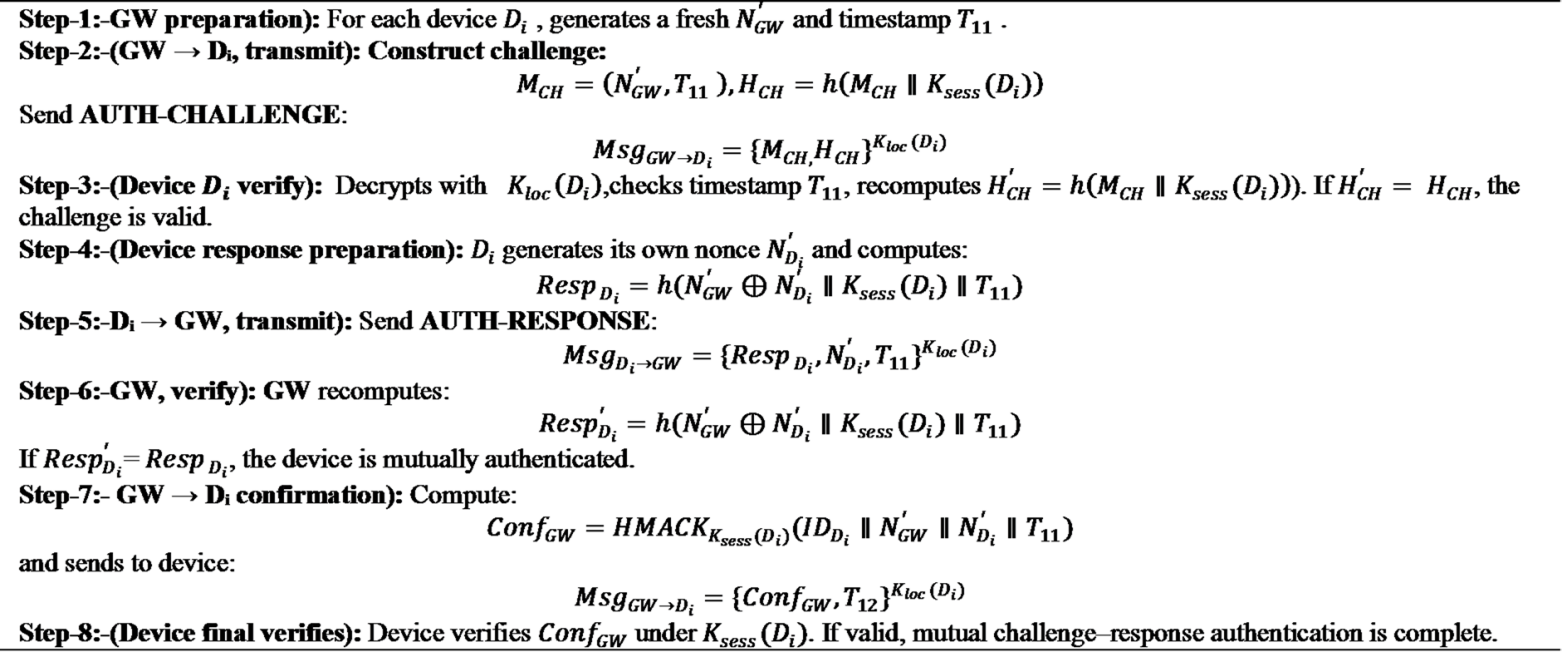
**Algorithm-1.9** AUTH-CHALLENGE/AUTH-RESPONSE (GW ↔ Device $\:{D}_{i}$​)-Phase 5 (Lightweight only).

And finally, in Algorithm-1.10, this phase installs an application session key per device and delivers a user-scoped access token. Using the session key negotiated in Phase 4, $\:{K}_{sess}\left({D}_{i}\right)$, the gateway derives the device’s application key $\:S{K}_{{D}_{i}}$ via a KDF that binds the device identity and a fresh timestamp. The key package $\:(S{K}_{{D}_{i}}\:,lifetime,{ctr}_{0},{T}_{13})$ is sent to the device over the symmetric transport $\:{K}_{loc}\left({D}_{i}\right)$, and authenticated with an HMAC under $\:{K}_{sess}\left({D}_{i}\right)$. The device verifies, installs $\:S{K}_{{D}_{i}}$,and returns a SESSION-READY acknowledgment HMAC under $\:S{K}_{{D}_{i}}$. After verifying the ack, the gateway derives a user token $\:{AT}_{U}$ from the login token $\:{ST}_{U}$​ and delivers it to the user, protected under a key derived from $\:{ST}_{U}$​. No ECC appears here; all work is hash/HMAC/KDF plus symmetric wrapping.

**Figure:**
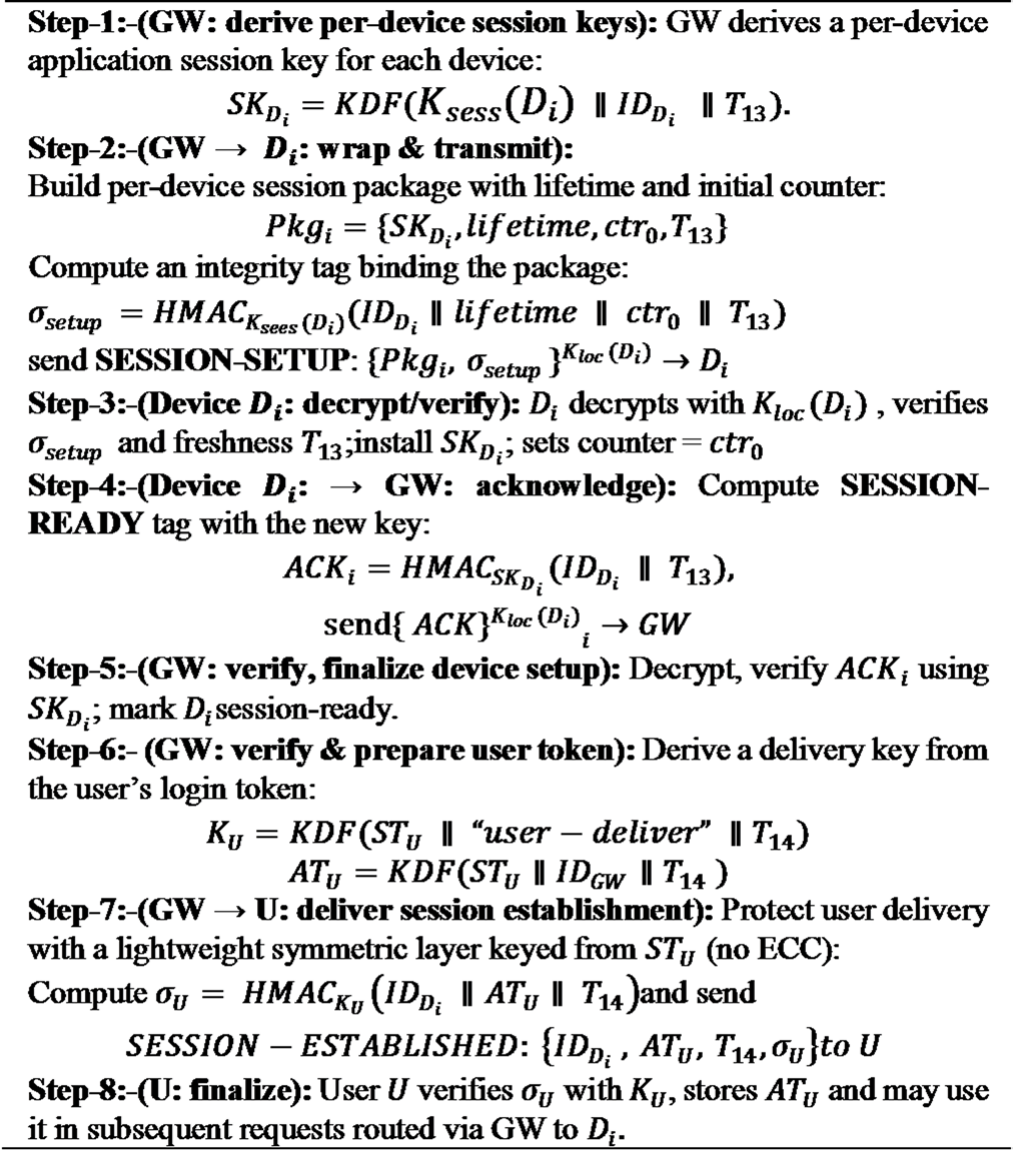
**Algorithm-1.10** Secure-session-EST & key-distribution (GW↔ Devices $\:{D}_{i}$; GW ↔ U)-Phase 6 Lightweight only; uses the per-device session key $\:{K}_{sess}\left({D}_{i}\right)$from Alg. 1.8; no ECC, no group semantics.

**Fig. 3:**
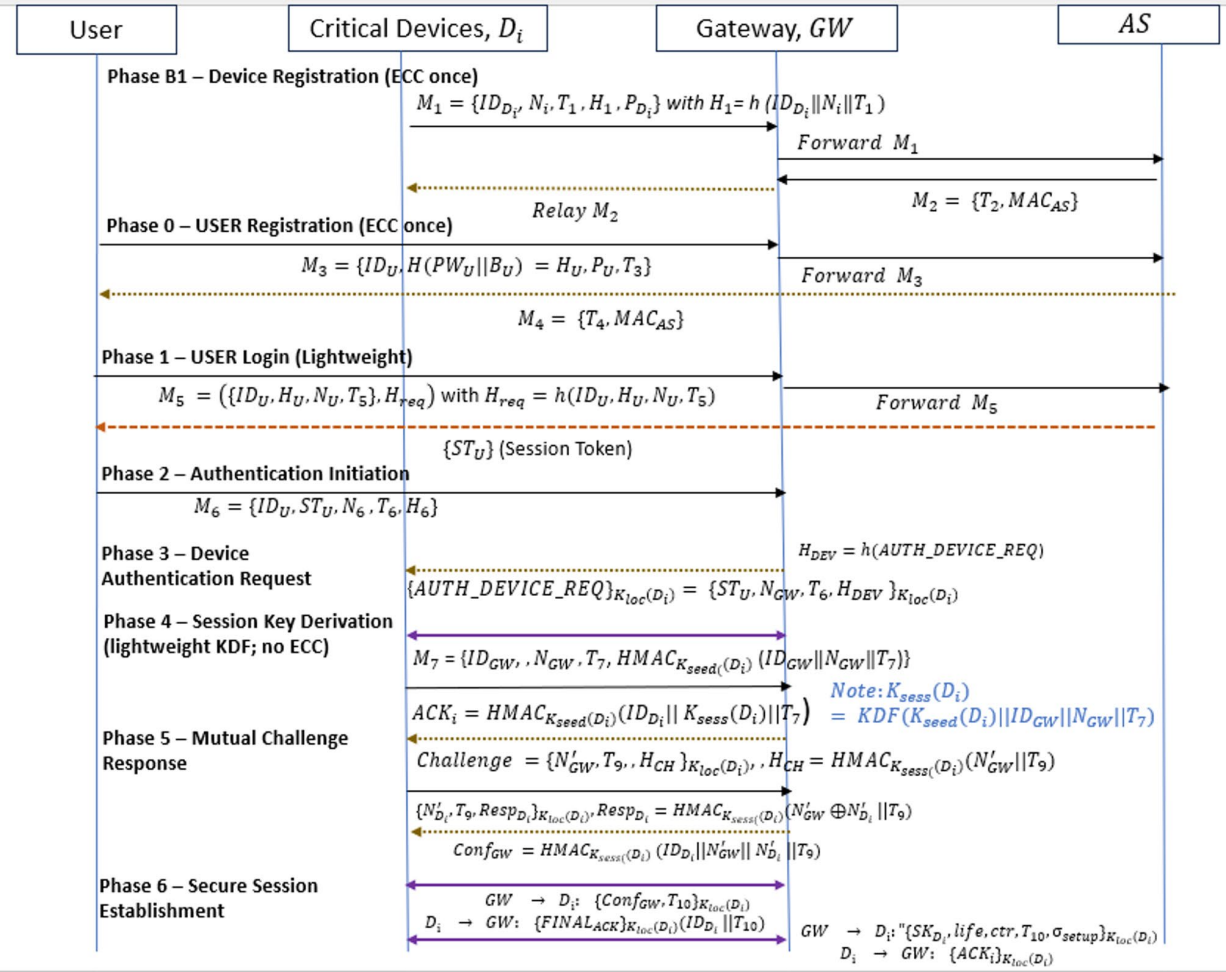
Message timing diagram of the proposed SELAM protocol across its phases.

Figure 3 shows the timing diagram that represents the complete message flow of during the authentication process between $\:U$, Critical Devices $\:{D}_{i}$ $\:,GW$ and $\:AS$. The figure illustrates the end-to-end authentication flow of the proposed Selective Elliptic Curve and Lightweight Authentication Mechanism (SELAM) for IoMT environments. The protocol is structured into ten sequential phases, beginning with device registration (Phase B1) and user registration (Phase 0), where ECC operations are performed only once to establish cryptographic credentials. All online phases (1–6) use lightweight primitives, hash/HMAC with timestamp freshness and device-local symmetric keys to minimize computation. Subsequent phases, including user login (Phase 1), authentication initiation (Phase 2), and device authentication request (Phase 3), employ lightweight primitives such as hashing, XOR, and timestamp validation to minimize computational cost. Session key derivation (Phase 4) leverages a KDF-based mechanism using long-term pre-established seeds, thereby avoiding repeated ECC operations in the online path. Mutual challenge–response validation (Phase 5) ensures freshness and authenticity, while secure session establishment (Phase 6) finalizes the process through the handshake with a gateway confirmation and device acknowledgment authenticated by HMAC, thereby establishing the secure session. By combining selective ECC usage during critical registration phases with lightweight operations during online authentication, SELAM achieves mutual authentication, replay resistance, and secure session key distribution with significantly reduced computational and communication overhead compared to Heavy+Verify ECC-per-device protocols.

## Mathematical analysis

In the proposed SELAM authentication protocol, entities include the User ($\:U$), Medical Device ($\:{D}_{i}$), Gateway (GW), and the Authentication Server (AS). Three categories of cost are studied:

1. Communication Cost – the number of bits exchanged in authentication messages^3^.
2. Computational Cost – the total number of cryptographic operations required^41^.
3. Authentication Overhead – the combined effect of communication and computation before secure data transfer begins^42^.

We instantiate the formulas in Section IV with empirically measured crypto timings and NS–3–derived payload sizes, ensuring numerical consistency between the analytical and empirical results.

### Communication cost

We compute communication cost by field accounting: each message is written as a concatenation of named fields, and the message cost is the sum of their bit-lengths; phase- and scheme-level costs are sums over messages in the corresponding phases (as in prior IoT/IoMT works)^21,43–45^. We have adopted a standardized 16-byte (128-bit) field profile for online communication accounting to ensure comparability across schemes. Thus, for the online path we account $\:\left|ID\right|=\:\left|N\right|=\:\left|T\right|=\left|H\right|=\:\left|MAC\right|=\left|ST\right|=\:\left|life\right|=\left|ctr\right|=$ 128 bits per field. $\:\left|K\right|$ is accounted as 128 bits only when it is transmitted as a wrapped key token (e.g., in Message-8 key setup). ECC artifacts (public keys/signatures) occur only during registration (Phases B1 and 0) and are excluded from online communication totals; they are accounted separately where registration costs are reported. ECC artifacts (public keys/signatures) occur only during registration (Phases B1 and 0) and are excluded from the online communication totals; they are accounted separately where registration costs are reported. For registration-only ECC provisioning, we instantiate ECC using a 256-bit curve (e.g., NIST P-256) to meet modern security strength expectations.

#### Message‑1 (Device Registration Request, $\:{\boldsymbol{D}}_{\boldsymbol{i}}\to\:\boldsymbol{G}\boldsymbol{W}\to\:\boldsymbol{A}\boldsymbol{S}$)

The device transmits its identity, nonce, timestamp, hash value, and ECC public key. The gateway forwards this to AS. Hence, the communication cost is.

1 $$\:{\:\:\:\:C\:}_{M1}=\:\left|ID\right|\:+\:\left|N\right|\:+\:\left|T\right|\:+\:\left|H\right|\:+\:\left|P\right|$$

#### Message‑2 (Device Registration ACK, $\:\boldsymbol{A}\boldsymbol{S}\to\:\boldsymbol{G}\boldsymbol{W}\to\:{\boldsymbol{D}}_{\boldsymbol{i}}$)

AS returns an acknowledgement including a timestamp authenticator and MAC. The gateway relays this to the device. The communication cost is.

2 $$\:{\:\:\:\:C\:}_{M2}\:=\:\left|T\right|\:+\:\left|MAC\right|$$

#### Message‑3 (User Registration, $\:\boldsymbol{U}\to\:\boldsymbol{G}\boldsymbol{W}\to\:\boldsymbol{A}\boldsymbol{S}$)

The user submits ID, hashed credentials, ECC public key, and timestamp. GW forwards this to AS. The communication cost is.

3 $$\:{\:\:\:\:C\:}_{M3}\:=\:\left|ID\right|\:+\:\left|H\right|\:+\:\left|P\right|\:+\:\left|T\right|\:$$

#### Message‑4 (User Registration ACK, $\:\boldsymbol{A}\boldsymbol{S}\to\:\boldsymbol{G}\boldsymbol{W}\to\:\boldsymbol{U}$)

AS confirms registration with a timestamp and MAC. The communication cost is.

$$\:{\:C\:}_{M4}=\:\left|T\right|\:+\:|MAC$$| (4).

#### Message‑5 (User Login$\:\to\:\boldsymbol{A}\boldsymbol{S}$, $\:\boldsymbol{U}\to\:\boldsymbol{G}\boldsymbol{W}\to\:\boldsymbol{A}\boldsymbol{S}$)

U sends identity, hashed credentials, nonce, timestamp, and login hash. GW forwards this to AS, which responds with a session token. The communication cost is.

5 $$\:{\:\:\:\:C\:}_{M5}\:=\left(\left|ID\right|+\:\left|H\right|+\:\left|N\right|+\:\left|T\right|\right)+(\left|ST\right|+\:\left|T\right|+\:\left|MAC\right|)$$

#### Message‑6 (Authentication Initiation, $\:\boldsymbol{U}\to\:\boldsymbol{G}\boldsymbol{W}$)

U transmits ID, session token, nonce, timestamp, and request hash. GW relays to AS. The communication cost is.

6 $$\:{\:\:\:\:C\:}_{M6}\:=\:\left|ID\right|\:+\:\left|ST\right|\:+\:\left|N\right|\:+\:\left|T\right|\:+\:\left|H\right|\:\:\:$$

**Message‑7 (Device Authentication and Session-KDF handshake**, $\:\boldsymbol{G}\boldsymbol{W}\leftrightarrow\:{\boldsymbol{D}}_{\boldsymbol{i}}$): $\:{C\:}_{M7}\:$combiness two lightweight rounds after user login: (1) the gateway sends a per-device auth request carrying the user token $\:{ST}_{U}$, a gateway nonce, and a timestamp with a hash tag; the device replies with a response MAC that binds both nonces and the timestamp, proving liveness and tying the request to the logged-in user; and (2) a tiny KDF pre-handshake where GW sends $\:({ID}_{GW},{N}_{GW})$ with an HMAC under the device’s registration seed $\:{K}_{seed}({D}_{i}$) and the device returns ($\:{ID}_{{D}_{i}}$) with its HMAC after locally deriving the same inputs, which “locks in” the Phase-4 per-device session key$\:{\:K}_{sess}({D}_{i}$). The communication cost is:

7 $$\begin{aligned}{C\:}_{M7}\:&=\:\left|ST\right|+\:\left|N\right|+\:\left|T\right|+\:\left|H\right|\:+\left|MAC\right|+\left|N\right|+\left|T\right|\nonumber\\&\quad +\left|ID\right|+\left|N\right|+\left|MAC\right|\:\:+\left|ID\right|+\left|MAC\right|\end{aligned}$$

**Message‑8 (Mutual Challenge–Response and Session Establishment**, $\:\boldsymbol{G}\boldsymbol{W}\leftrightarrow\:{\boldsymbol{D}}_{\boldsymbol{i}}$**)**:

GW sends a challenge (its ID, session token, fresh gateway nonce, timestamp, challenge hash). The device replies with its ID, the same token, a fresh device nonce, its timestamp, and a MAC binding both nonces and the timestamp. After verification, GW transmits a key-setup package containing its ID and token plus a wrapped application key, lifetime, counter, setup timestamp, and the AEAD nonce and tag. The device returns a ready message (ID, token, MAC); GW sends a short final-ACK (token, MAC); the device finishes with a confirm MAC. The communication cost is:

8 $$\begin{aligned}\:{\:\:\:\:C\:}_{M8}&=\:(\left|ID\right|+\left|ST\right|+\left|N\right|+\:\left|T\right|+\:\left|H\right|)+(\left|ID\right|+\left|ST\right|+\left|N\right|+\:\left|T\right|+\:\left|MAC\right|)\nonumber\\ &\quad +(\left|ID\right|+\left|ST\right|+\left|K\right|+\left|life\right|+\left|ctr\right|+\left|T\right|+\:\left|N\right|+\:\left|MAC\right|)\nonumber\\ &\quad +(\left|ID\right|+\left|ST\right|+\left|MAC\right|)+\left(\left|ST\right|+\left|MAC\right|\right)+(\left|MAC\right|)\end{aligned}$$

Thus, the online per-round communication cost (runtime) is:

9 $$\:{\boldsymbol{C}}_{\boldsymbol{o}\boldsymbol{n}\boldsymbol{l}\boldsymbol{i}\boldsymbol{n}\boldsymbol{e}}^{\boldsymbol{S}\boldsymbol{E}\boldsymbol{L}\boldsymbol{A}\boldsymbol{M}}=\:\:\sum\:_{\boldsymbol{i}=5}^{8}\left|{{\boldsymbol{C}\:}_{\boldsymbol{M}}}_{\boldsymbol{i}}\right|\:$$

### Computational cost

In the proposed protocol, asymmetric operations (ECC) are invoked selectively, while lightweight operations (hash, XOR, timestamp validation) dominate the online authentication path. Specifically, the ECC operations occur only in Phase B1 (Device Registration: ECC key generation & public key transmission) and Phase 0 (User Registration: ECC key generation for user). No ECC is executed online; Phase 4 derives a per-device session key derivation via KDF. The hash operations protect nonces and timestamps in login, challenge–response, and session establishment (Phases 1, 5, 6). XOR operations are used for lightweight key derivation and verification, and timestamp checks provide freshness guarantees in all transmitted messages. Let $\:{O}_{ECC}$,$\:{O}_{h}$, $\:{O}_{XOR}$, $\:{O}_{TS}$, denote unit operation costs. The total computational cost of SELAM over one full authentication cycle is:

10 $$\:{C}_{comp}^{SELAM}=\:\alpha\:{O}_{ECC}+\beta\:{O}_{h}+\gamma\:{O}_{XOR},\:+\:{\updelta\:}{O}_{TS},\:\mathrm{w}\mathrm{i}\mathrm{t}\mathrm{h}\:\alpha\:=0\:\:\:\:\:\:\:\:\:\:\:\:\:\:$$

where:

- $\:\alpha\:$ = number of ECC operations.
- $\:\beta\:$= number of hash operations (across login and challenge–response).
- $\:\gamma\:$= number of XOR operations (used in key updates).
- δ = number of timestamp validations (distinct freshness checks across the round).

### Authentication overhead

The end-to-end overhead before secure data transfer is:

11 $$\:{O}_{auth}=\:{C}_{online}^{SELAM}+{C}_{comp}^{SELAM}$$

### Empirical analysis

This section evaluates the empirical performance of the proposed SELAM protocol. The analysis instantiates the mathematical equations derived in Section III using standardized credential sizes and benchmarked cryptographic operation costs. Following established IoT/IoMT communication-cost reporting by message-field accounting, we adopt a standardized 16-byte (128-bit) field profile for online messages to enable fair cross-scheme comparison^35^. Under this profile, online fields such as identifiers, nonces, timestamps, hashes/MACs, counters, and wrapped keys are accounted as 16 bytes each in the online communication-cost calculation. In SELAM, ECC artifacts (e.g., public keys or signatures) occur only during registration (Phases B1 and 0) and are therefore excluded from online totals; registration costs are reported separately. For the signature-based baseline, we size messages via the same field-accounting method^22^ using its own field set. For SELAM, the number of cryptographic fields per message is taken directly from our protocol design, which confines ECC usage to the registration phases only (B1 and 0); Phase 4 uses a KDF (no ECC) for per-device session key derivation, while relying on lightweight primitives (hash, XOR, timestamp) in the online path. For the baseline ECC-per-device comparator, we model a signature-based mutual-authentication design in which messages carry a verifiable credential (e.g., certificate/EC public key) and an ECDSA-class signature; such per-message signature AKES are standard in the IoT literature, and their communication accounting follows fixed field sizes^35,36^. Under these standardized assumptions, the per-device communication and computation costs reported in Tables 1, 2 and 3 are directly comparable and reproducible.

Let $\:m\:$denote the number of devices for which Message-7 (per-device device-auth + KDF pre-handshake) executes within a round. Messages 5, 6 and 8 are one-per-round. Substituting these values into Eq. (9), for a single critical device ($\:m=1$), the total communication cost of SELAM per authentication cycle is obtained as:

$$\:{C}_{online}^{SELAM}=\left(112+80+384\right)+192m=576+192m\:bytes$$

and for single device per-round:

$$\:{C}_{online}^{SELAM}\sum\:_{i=5}^{8}\left|{{C\:}_{M}}_{i}\right|\:\mathrm{f}\mathrm{o}\mathrm{r}\:\:\:\left(\mathrm{m}=1\right)=576+192=768\:bytes=6144\:bits$$

**Table 3:** Communication cost of SELAM.

Protocol	Total communication (bytes)	Total communication (bits)
Baseline ECC	960	7680
SELAM online	768	6144

In SELAM, ECC is invoked only during device and user registration (two scalar multiplications in total); all online phases use lightweight primitives (HMAC/AEAD, hash, XOR, timestamps) only. In the per-device online cycle, this results in $\:\alpha\:=0$ ECC operations and, depending on accounting, $\:\beta\:=9$ hash-equivalents, γ = 2 XORs, and δ = 7 timestamp checks. The empirical tables adopt conservative accounting which matches our simulator’s fast path and keeps parity with standardized reporting in related work. From Eq. (10), the computational cost for SELAM is:

$$\:{C}_{comp}^{SELAM}\text{}\approx\:9\left(0.01\right)+2\left(0.001\right)+7\left(0.001\right)\approx\:0.10\:\mathrm{m}\mathrm{s}$$

*(Using the standardized timings*: $\:{O}_{ECC}=2.94\:ms$, $\:{O}_{h}=0.01\:ms$, $\:{O}_{XOR},{O}_{T}<0.001\:ms$*)*.

For comparison, a baseline ECC-per-device scheme requires $\:m$ ECC operations in the online path ($\:\alpha\:=m$), yielding (Table 4):

$$\:{C}_{comp}^{baseline}\approx\:8\left(2.94\right)+0.071\approx\:23.59\mathrm{m}\mathrm{s}$$

**Table 4:** Computation cost comparison.

Protocol	ECC ops	Hash ops	XOR ops	Timestamp ops	Total time (ms)
Baseline ECC	8	6	2	7	23.59
SELAM	0	9	2	7	0.10

And finally, Authentication overhead is defined as the sum of communication and computation costs (Eq. 11). To express both in units of time, communication is converted to transmission time using a typical IoMT link rate. For Bluetooth Low Energy (BLE) at 1 Mb/s, the communication delay is (Table 5):

- SELAM: $$\:6144/{10}^{6}=6.144\:\boldsymbol{m}\boldsymbol{s}$$
- Baseline: $$\:7680/{10}^{6}=7.680$$ ms.

**Table 5:** Authentication overhead comparison.

Metrics	Baseline ECC	SELAM
Communication	7.680 ms	6.144 ms
Computation	23.59 ms	0.100 ms
$\:{\boldsymbol{O}}_{\boldsymbol{a}\boldsymbol{u}\boldsymbol{t}\boldsymbol{h}}$	31.27 ms	6.244 ms
Overhead trend	Linear in ECC ops	Constant ECC, Lightweight

Under the standardized 16-byte credential profile, SELAM reduces per-device communication by 20% (from 960 B to 768 B) and computation time by ≈approximately 99% (from 23.6 ms to 0.10 ms) relative to a baseline ECC-per-message scheme. These gains stem from SELAM’s selective use of ECC invoked only during registration, while the online path relies on lightweight primitives (hash/HMAC, XOR, timestamp). AEAD is employed for the confidentiality and integrity of keying material; its CPU cost is negligible at this scale (≈ 0.02 ms if explicitly accounted for) and does not alter the conclusion. Consequently, SELAM is a practical authentication framework for resource-constrained critical IoMT devices, delivering lower overhead without weakening security guarantees.

## Performance analysis

In this section, we present comprehensive simulation results to prove SELAM’s performance and security effectiveness. We evaluate SELAM under a single-hop IoMT topology (device to gateway to authentication server). ECC is confined to registration only with Phase B1 (device) and Phase 0 (user), while all in-session authentication uses lightweight operations (hash/XOR/timestamp) to reduce ongoing authentication cost. Experiments use $\:n=136\:$devices, header-inclusive accounting, and two scenarios: no-attack and with-attack (impersonation/replay traffic present during authentication). Distributional statistics (median and $\:{p}_{90}$) are used to reduce sensitivity to outliers.

We evaluate SELAM against a Heavy+Verify baseline that performs online ECC verification per session. We report authentication success outcomes under attack injection and quantify overhead using (i) end-to-end authentication delay, (ii) computation time breakdown per online phase, and (iii) communication cost using standardized field accounting. To reduce sensitivity to outliers, we report distributional statistics (e.g., median and tail percentiles) and aggregate results across multiple random seeds where applicable.

### Experimental setup and dataset mapping

#### Dataset-driven 10-phase emulation and attack injection

CICIoMT-2024 logs are used to drive workload intensity and session composition, not to supply cryptographic tokens. Each log entry is mapped to one protocol run by assigning dataset-level communication events to SELAM protocol entities (device, gateway, authentication server) and executing the SELAM phase sequence. Phases A/B1/0 generate setup and registration materials once per principal (ECC keys and long-term seeds), while Phases 1–6 execute per session. Fields not present in the dataset (nonces, MAC tags, challenge–response values, and derived session keys) are generated by the simulator using cryptographically secure randomness and the specified primitives.

Workload intensity is controlled by varying the number of concurrent authentication sessions (*N* = 136–2000). Benign dataset traces correspond to valid authentication sessions, while adversarial conditions are introduced by transforming the message stream. Replay attacks are simulated by resending previously emitted messages outside the freshness window Δ, while impersonation and MitM attacks attempt to substitute identities and forge Phase-5 validation tokens. DoS behavior is emulated by increasing concurrent authentication load. This separation explicitly distinguishes dataset-driven session traces (workload and traffic patterns) from protocol-generated cryptographic artifacts.

All simulations are conducted using a Python-based discrete-event simulation framework on a system equipped with an 11th Gen Intel Core i5-1135G7 processor (2.40 GHz), 8 GB RAM, running Microsoft Windows 10 Home (Build 19045), with Python version 3.12.3.

And for reproducibility, all results are averaged over 20 independent simulation seeds. Reported statistics (e.g., median, percentile values) ensure robustness against outliers. To reproduce the reported results, the evaluation follows a consistent pipeline: (i) preprocess CICIoMT-2024 logs to extract session-level traces, (ii) map each trace to a SELAM protocol execution as described above, (iii) generate cryptographic artifacts (nonces, MACs, session keys) using the simulator, (iv) inject adversarial behaviors (replay, impersonation, MitM, DoS) according to the defined threat model, (v) execute simulations across varying cohort sizes (*N* = 136–2000) and network parameters, and (vi) compute performance and security metrics (delay, communication cost, ASR, FAR/FRR) aggregated over 20 independent seeds.

### End-to-end authentication delay (latency)

**Fig. 4:**
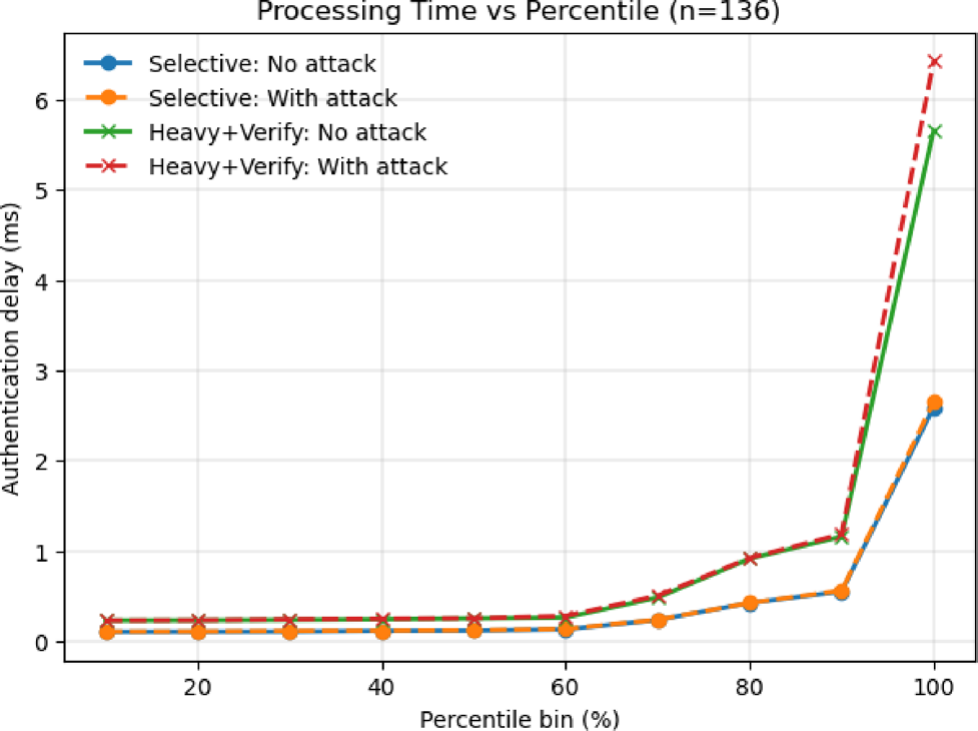
End-to-end authentication delay vs. number of devices/sessions for SELAM.

Figure 4 depicts the distribution of per-device authentication delay. Across the entire range from the 10th to the 95th percentile, the SELAM curve lies strictly below Heavy+Verify, and the separation widens towards the upper tail. Using the 90th percentile $\:{p}_{90}$ as the summary statistic, SELAM reduces end-to-end latency from 1.226 ms to 0.652 ms in the benign regime (a 46.8% improvement) and from 1.170 ms to 0.515 ms under attack (a 56.0% improvement). These improvements follow directly from removing ECC verification from the online authentication path (the performance-critical steps executed every session). Under attack, the Heavy+Verify performs more verifications, so its delay grows while SELAM avoids that cost, making the gap larger.

#### Impact of network bandwidth on authentication delay

To evaluate the effect of heterogeneous IoMT network conditions, authentication delay is analyzed across multiple representative link rates (250 kb/s, 500 kb/s, 1 Mb/s, and 2 Mb/s). As expected, end-to-end delay scales inversely with available bandwidth, as communication latency is directly proportional to message size and transmission rate. This relationship can be approximated as:

$$\:Delay\:\left(D\right)\:=\:\frac{B\_total\:}{R}$$

where $\:{B}_{total}$ is number of transmitted bits and $\:R\:$is link bandwidth. Despite this variation in absolute delay, SELAM consistently maintains lower communication overhead compared to the baseline due to reduced message sizes and fewer exchanges per authentication session. This results in a stable relative performance advantage across all bandwidth settings. In low-bandwidth IoMT scenarios, where communication cost becomes a dominant factor, the lightweight design of SELAM further reduces transmission delay and improves reliability, demonstrating its suitability for resource-constrained medical environments.

### Communication cost and scalability

We report (i) protocol-level field-accounting bits/device and (ii) ns-3 header-inclusive bits/device. The latter is larger due to network headers, but both measurements preserve the same qualitative conclusion: SELAM < Heavy+Verify and the scaling direction with $\:\boldsymbol{N}$ is consistent.

**Fig. 5:**
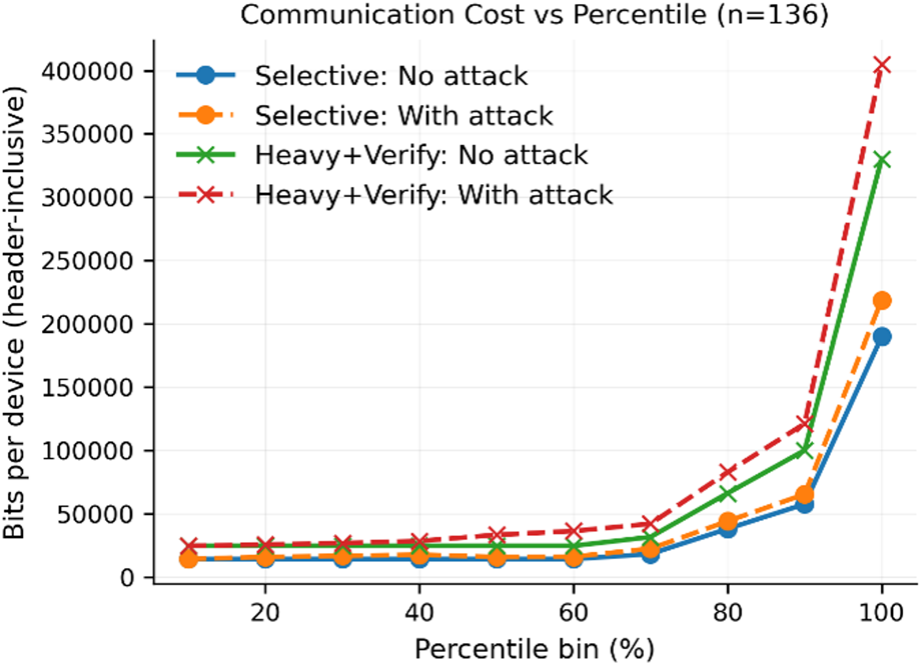
SELAM communication cost vs. percentile (header-inclusive).

Figure 5 examines the communication cost per device with header-inclusive accounting. The SELAM curve again remains below across deciles in both regimes. Averaged over devices, the benign runs show 39,416 bits for SELAM versus 45,703 bits for Heavy+Verify (a 13.8% reduction), while the attack runs yield 39,831 bits versus 46,470 bits (a 14.3% reduction). These measured savings are directionally consistent with the analytical payload-only calculation (6144 vs. 7680 bits, i.e., − 20%): fixed link/network headers and hop replication in the simulator dilute the payload advantage, but the ordering and the magnitude remain aligned with the model.

**Fig. 6:**
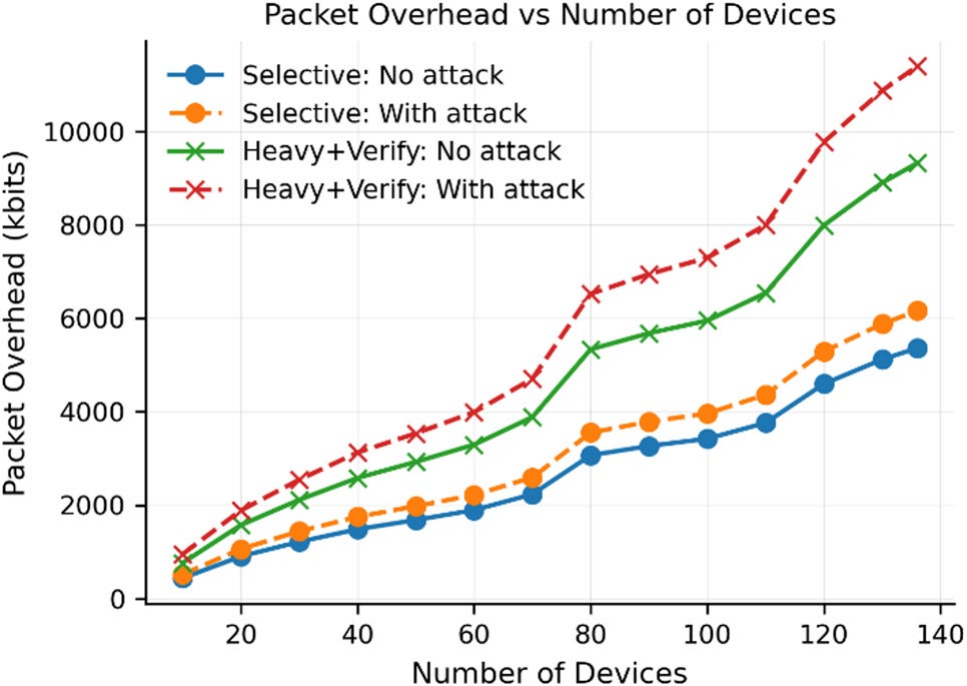
The proposed SELAM Packet overhead vs. the number of devices.

Figure 6 studies scalability with cohort size by plotting cumulative header-inclusive overhead as the device set grows. Both schemes scale roughly linearly with $\:N$, but once $\:N$ exceeds roughly 80–100 devices, the SELAM curve remains strictly below the Heavy+Verify across the measured range, with a visibly larger separation once $\:N$ exceeds roughly 80–100 devices. This behaviour is consistent with per-session savings that accumulate across the cohort; it complements the per-device perspectives of Figs. 5 and 6.

### Cryptographic computation time

**Fig. 7:**
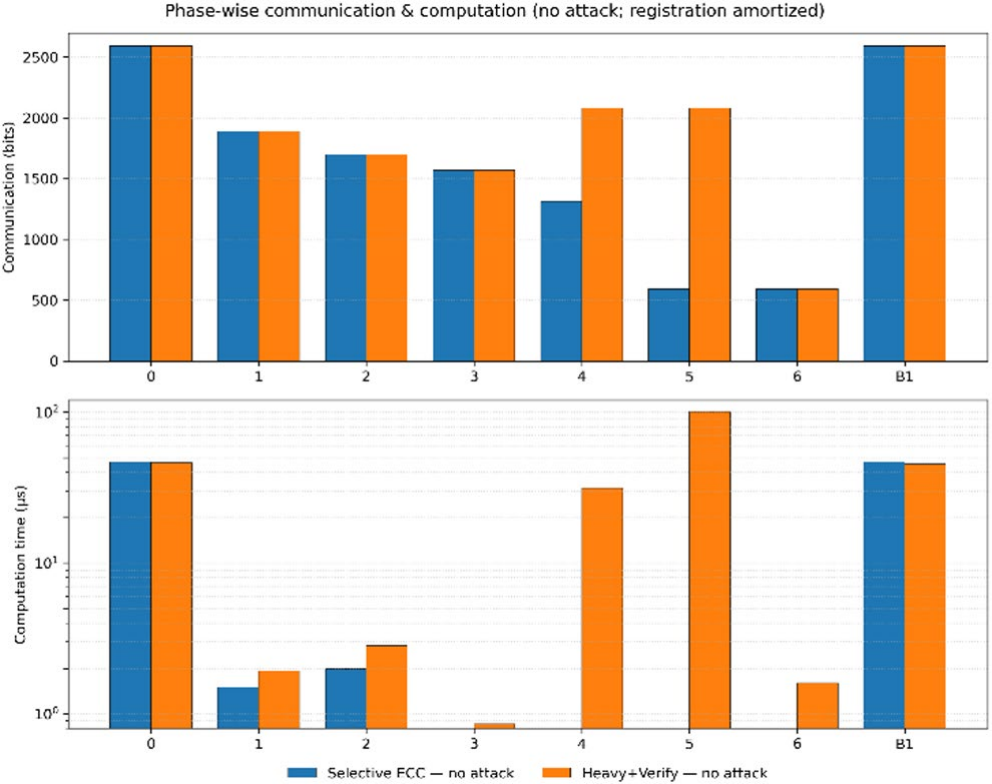
SELAM phase-wise communication and cryptographic computation under normal load.

To determine whether the savings are due to cryptographic path design rather than network artefacts, we instrument per-phase computation. Figure 7 breaks down, by phase, the communication (top panel, bits) and compute time (bottom panel, $\:\mu\:s$, log scale) under the no-attack condition. One-time registration phases (B1 and 0) are shown for completeness but are amortized in the totals. Across the online phases (1–6), SELAM is dominated by lightweight primitives (hash/HMAC, XOR, timestamp checks). Its per-phase compute stays sub-millisecond, and its message sizes are consistently smaller in Phases 4 to 6. By contrast, Heavy+Verify shows clear spikes, most notably in Phase 4 (key establishment/verification) and Phase 5 (the online ECC verify) because it executes ECC at runtime. The registration phases (B1, 0) look similar for both schemes, as both perform ECC during enrolment. This pattern is exactly what SELAM is designed to achieve: confine ECC to registration and keep the online path lightweight. The phase trace, therefore, attributes the observed latency and bandwidth gains to cryptographic design rather than network effects, and it is consistent with the unchanged ASR under attack.

Sections A–C report distribution-level behavior for the baseline cohort (*n* = 136) and small-N trends, while Section D extends the evaluation to large-scale adversarial load (*N* = 136–2000) using the same acceptance logic and fixed attack probabilities.

### Reliability under adversarial load (ASR)

**Fig. 8:**
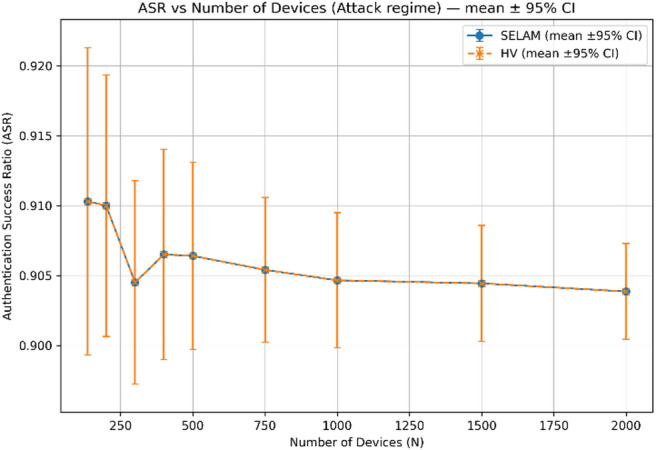
The proposed SELAM authentication success ratio (ASR vs. $\:\boldsymbol{N}$ (attack) multi-seed mean ± 95% CI.

We evaluate reliability under a fixed attack regime with per-device replay probability $\:{\boldsymbol{p}}_{\boldsymbol{r}}=0.05$ (Phase 3) and impersonation probability $\:{\boldsymbol{p}}_{\boldsymbol{i}}=0.05$ (Phase 5); when an attack is triggered, one additional forged/replayed attempt is injected. All reported means and 95% CIs are computed over multiple RNG seeds, while keeping $\:{\boldsymbol{p}}_{\boldsymbol{r}}$and $\:{\boldsymbol{p}}_{\boldsymbol{i}}$constant.

We compute the Authentication Success Ratio (ASR) as the fraction of devices that complete an authentication session without any attack-induced termination markers. A device is counted as unsuccessful if its phase logs contain explicit drop or verification-failure markers (e.g., ReplayDrop, HMACVerify_Fail, ECCVerify_P256_Fail), which indicate that the protocol correctly rejected an adversarial attempt or that the session did not complete.

Figure 8 shows that ASR remains high and stable with scale (≈ 0.90–0.92 across $\:\boldsymbol{N}$) and that SELAM and Heavy+Verify exhibit essentially overlapping ASR across all tested cohort sizes. This overlap is expected because both schemes apply the same online acceptance policy under replay and impersonation; timestamp freshness checking and challenge–response verification, whereas SELAM differs primarily in how much cryptographic work is performed during the online phases. Therefore, selectively removing online ECC verification does not reduce correctness or acceptance reliability under the tested adversarial load, and the schemes are differentiated primarily by computational and communication overhead rather than ASR.

**Fig. 9:**
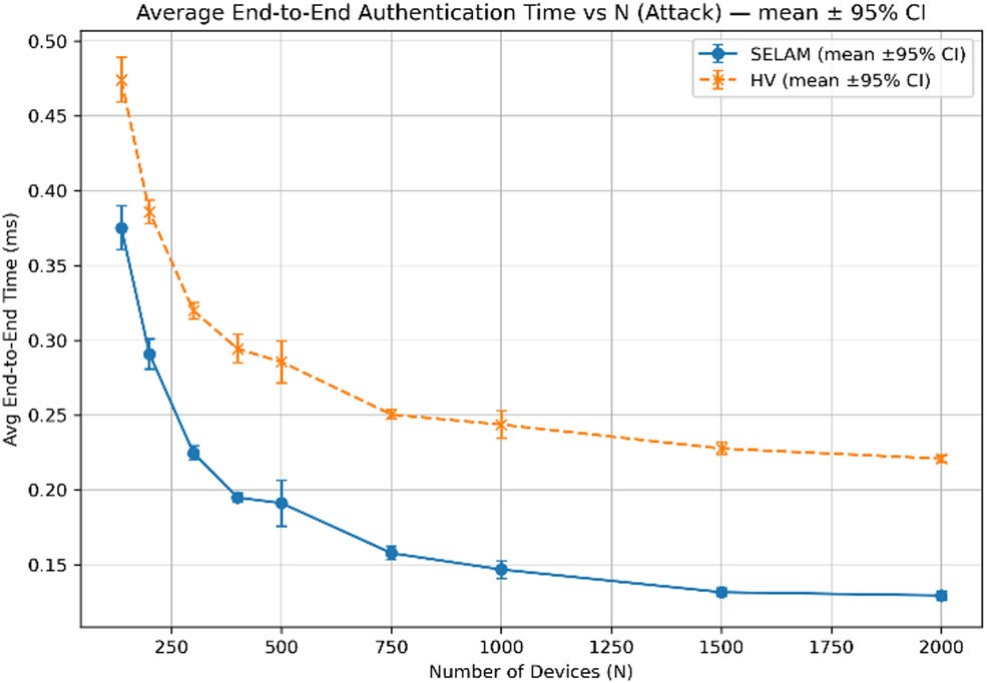
The proposed SELAM Average end-to-end authentication time vs. N (attack).

Figure 9 reports the average end-to-end authentication time under the same attack profile. SELAM consistently achieves lower latency than Heavy+Verify as N increases. The mechanism is structural: Heavy+Verify performs online ECC verification in the critical online path (Phase 5), whereas SELAM confines ECC to provisioning/registration and relies on lightweight online validation (hash/XOR/timestamp checks). Consequently, SELAM’s online runtime remains dominated by symmetric operations, leading to lower end-to-end delay under attack and at scaling.

Table 6 reports the header-inclusive online communication overhead (bits/device) under attack. Two observations follow. First, overhead is approximately constant across N, because the message field profile is fixed and the attack injection rates are fixed (thus, (Phase 3) the expected number of extra attempts does not grow with N). Second, SELAM achieves a consistent communication reduction of ~ 32% relative to Heavy+Verify across all N (e.g., ≈ 14.25k bits/device vs. ≈ 21.0k bits/device), indicating that removing online ECC verification artifacts and associated payload inflation translates directly into lower transmitted bits per device under adversarial traffic.

**Table 6:** Average online communication overhead per device (bits/device) under attack regime across cohort size $\:N$ (mean ± 95% CI over 20 seeds).

$\:\boldsymbol{N}$	SELAM bits/device (mean ± 95% CI)	Heavy + verify bits/device (mean ± 95% CI)	Reduction (%)
136	14241.47 ± 11.85	20993.24 ± 37.20	32.16
200	14243.70 ± 12.21	20987.81 ± 23.75	32.13
300	14248.41 ± 9.73	21005.80 ± 19.83	32.17
400	14248.65 ± 8.49	20995.02 ± 22.18	32.13
500	14246.65 ± 6.40	21004.14 ± 20.29	32.17
750	14250.29 ± 6.60	21001.07 ± 14.17	32.14
1000	14249.48 ± 6.05	21006.22 ± 13.57	32.17
1500	14250.24 ± 4.85	21005.53 ± 12.54	32.16
2000	14249.18 ± 4.27	21008.78 ± 9.06	32.18

Selam reduces payload by ~ 32.1–32.2% versus Heavy+Verify, and per-device overhead remain essentially constant with scaling due to fixed FIELD SIZES per session.

While ASR captures overall authentication success under adversarial conditions, protocol-level correctness is further quantified using FAR and FRR metrics, discussed in the next section.

### False acceptance rate (FAR) and false rejection rate (FRR)

To complement ASR-based reliability analysis, we define protocol-level False Acceptance Rate (FAR) and False Rejection Rate (FRR). We also report protocol-level false reject and false accept rates derived from the 20-seed PhaseLogs. We compute FRR as the fraction of benign authentication sessions that fail to complete the online pipeline (i.e., do not reach Phase 6). We compute $\:{\boldsymbol{F}\boldsymbol{A}\boldsymbol{R}}_{\boldsymbol{r}\boldsymbol{e}\boldsymbol{p}\boldsymbol{l}\boldsymbol{a}\boldsymbol{y}}$ and $\:{\boldsymbol{F}\boldsymbol{A}\boldsymbol{R}}_{\boldsymbol{i}\boldsymbol{m}\boldsymbol{p}}$ as the fraction of injected replay/impersonation attempts that nonetheless complete the online pipeline without triggering any logged rejection marker (e.g., ReplayDrop, HMACVerify_Fail, or integrity/freshness failures). Across *N* = 136–2000, we observe FRR = 0.000 in the benign regime and $\:{\boldsymbol{F}\boldsymbol{A}\boldsymbol{R}}_{\boldsymbol{r}\boldsymbol{e}\boldsymbol{p}\boldsymbol{l}\boldsymbol{a}\boldsymbol{y}}$ = 0.000, $\:{\boldsymbol{F}\boldsymbol{A}\boldsymbol{R}}_{\boldsymbol{i}\boldsymbol{m}\boldsymbol{p}}$ = = 0.000 under attack (mean ± 95% CI = 0.000 ± 0.000; all seeds yielded zero), indicating no accepted attacks and no benign rejections under the evaluated settings. Because SELAM uses deterministic cryptographic checks (MAC/AEAD + freshness), non-zero FAR/FRR would arise primarily from clock desynchronization, overly strict Δ, or implementation faults; under the evaluated Δ and network conditions, all benign sessions complete, and all injected replay/impersonation attempts are rejected.

### Parameter sensitivity analysis (freshness window $\:\boldsymbol{\varDelta\:}$ and nonce length$\:\:\left|\boldsymbol{N}\right|$)

We further analyze sensitivity to two parameters that directly affect correctness–security trade-offs and communication cost: the freshness window Δ used for timestamp acceptance, and the nonce length $\:\left|\boldsymbol{N}\right|$ used in challenge–response exchanges. Freshness checking introduces a direct trade-off between availability and replay resistance. If the acceptance window Δ is set too small, otherwise valid requests may be rejected because of normal clock drift and network delay; if Δ is set too large, delayed replayed messages are more likely to fall within the allowed window. Freshness-window sensitivity is evaluated via a controlled Monte-Carlo model of clock skew and replay arrival, while nonce-length sensitivity is computed by recomputing message sizes under the same field accounting model.

#### Freshness window $\:\boldsymbol{\varDelta\:}$

**Fig. 10:**
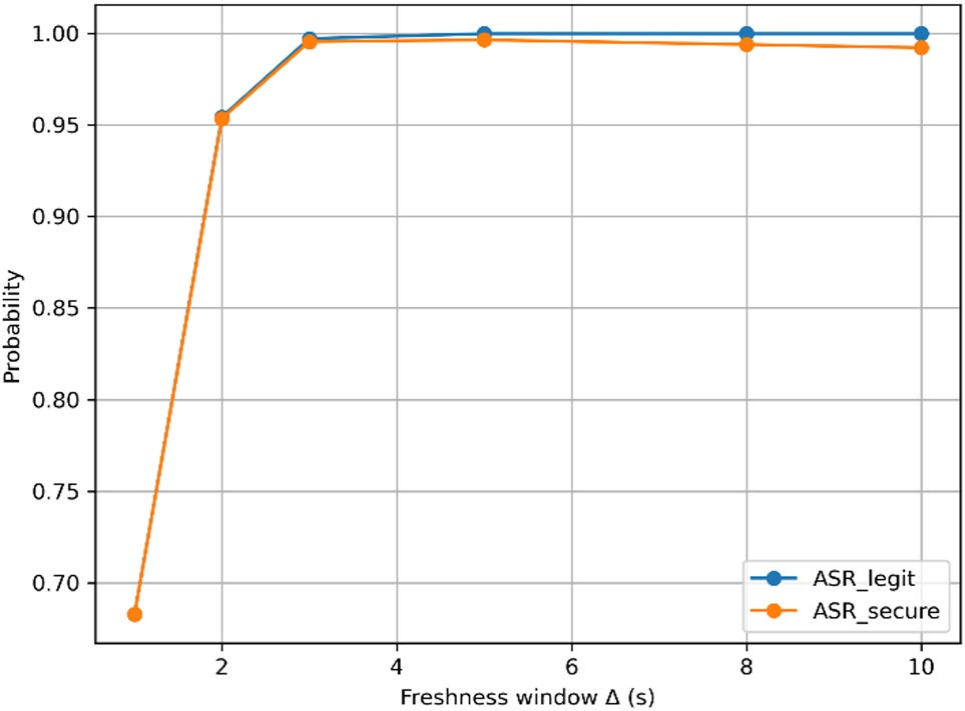
ASR vs. freshness window Δ.

As shown in Fig. 10, the legitimate acceptance rate (ASR_legit) improves rapidly as Δ increases. Under moderate clock skew (standard deviation ≈ 1 s), Δ = 1s results in low reliability (ASR_legit ≈ 0.68). Increasing Δ to 2s raises acceptance substantially (≈ 0.95), while Δ ≥ 3 s yields near-complete legitimate acceptance (≈ 0.997–1.0).

**Fig. 11:**
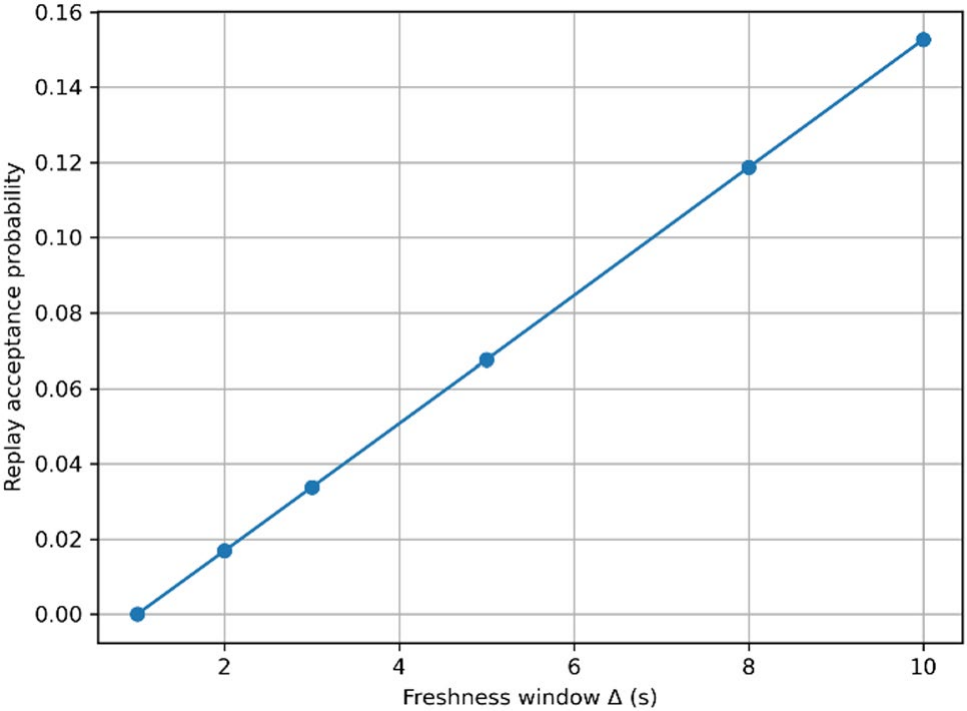
Replay acceptance probability vs. Δ.

This improvement comes with a cost. Figure 11 indicates that replay acceptance increases monotonically with Δ under the same skew setting: approximately 0.067 at Δ = 5 s and 0.152 at Δ = 10 s. Hence, Δ should be chosen deliberately to balance the risk of rejecting legitimate sessions against the risk of admitting replays. In this study, we adopt Δ = 5 s as a practical compromise that achieves near-unity legitimate acceptance while keeping replay acceptance at a bounded level.

#### NonceLength$\:\:\left|\boldsymbol{N}\right|\:$

**Fig. 12:**
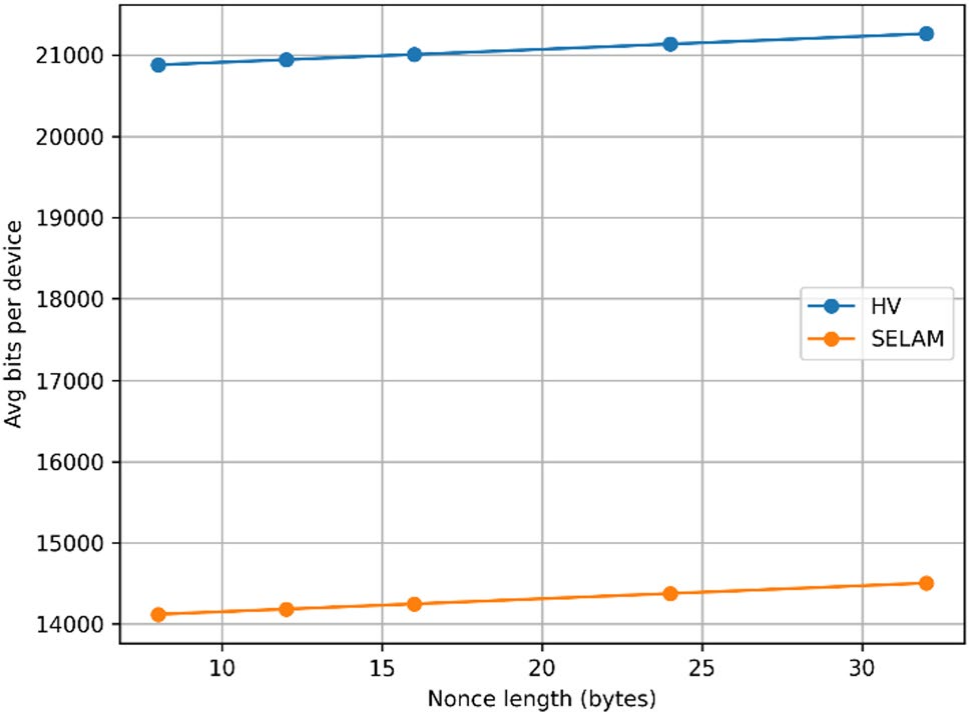
Nonce-length sensitivity: average bits/device vs. nonce length (bytes), $\:N=2000$

Figure 12 reports nonce-length sensitivity in terms of online communication overhead at $\:\boldsymbol{N}=2000$. As nonce length increases, the per-device bits increase approximately linearly for both schemes because the nonce field appears in the online challenge–response messages. Importantly, the absolute gap between SELAM and Heavy+Verify remains nearly constant across nonce lengths, indicating that SELAM’s advantage is structural (elimination of online ECC verification payload/overhead) rather than dependent on a particular nonce size. We therefore retain a 16-byte nonce as the default setting, which is sufficient for practical anti-replay uniqueness while avoiding unnecessary communication inflation.

### Summary of performance results

Across analysis and simulation, SELAM delivers substantial efficiency gains while preserving reliability. With 16-byte standardized credentials, per-session payload-only communication decreases from 960 B (Heavy+Verify) to 768 B (SELAM) (− 20%; 7,680 → 6,144 bits), and the corresponding transmit time at 1 Mb/s decreases from 7.680ms to 6.144ms. Eliminating online ECC verification reduces the analytical online computation cost from ≈ 23.59 ms/device to ≈ 0.10ms/device (− 99.6%) and yields an end-to-end authentication overhead of ≈ 6.24ms versus ≈ 31.27ms. In Python microbenchmarks (runtime phases only, *N* = 136), SELAM reduces the 90th percentile end-to-end authentication delay from 1.226ms to 0.652ms under benign traffic (− 46.8%) and from 1.170ms to 0.515ms under scripted attacks (− 56.0%). Under fixed replay and impersonation injection rates (*p* = 0.05 each), multi-seed scaling experiments (20 seeds) show that ASR remains stable with scale up to *N* = 2000 and that SELAM and Heavy+Verify exhibit overlapping ASR (mean ± 95% CI), so the observed gap (ΔASR ≲ 0.02) is not statistically meaningful; it is primarily driven by conservative early rejection under freshness/MAC failures rather than false acceptance. SELAM also reduces header-inclusive transmitted bits/device by ≈ 32% across all N (Table 6). Parameter sensitivity results clarify the operational envelope: the freshness window Δ controls the false-reject vs. replay-tolerance trade-off (Δ = 1 s is overly strict; Δ ≥ 3 s is reliable), while nonce length increases bits/device linearly without changing the qualitative gap between schemes (Table 7).

**Table 7:** Comparison consolidated performance summary.

Metric	Heavy+Verify ECC (per-device)	SELAM (per-device)	Improvement
Communication per round	960 B (7680 bits)	768 B (6144 bits)	−20%
Online computation time	23.59ms	0.10ms	−99.6%
Total auth overhead @ 1 Mb/s	31.27ms	6.24ms	−80%
Online ECC ops/device	8	0	eliminated
ASR (attack-only, 10–140 devices)	0.90–0.92	0.88–0.90	Δ ≤ ~0.02
p90 E2E latency (empirical, benign)	1.226ms	0.652ms	− 46.8%
p90 E2E latency (empirical, attack)	1.170ms	0.515ms	− 56.0%
Reliability under adversarial load (ASR, scaling *N* = 136–2000; mean ± 95% CI)	0.88–0.91	0.88–0.91	≈ 0 (overlapping CI)S
Freshness window Δ sensitivity (trade-off: availability vs. replay acceptance)	ASR_secure: 0.683 (Δ = 1s) → 0.996 (Δ = 3s); ReplayAccept: 0.000 (Δ = 1s) → 0.154 (Δ = 10s)	ASR_secure: 0.683 (Δ = 1s) → 0.996 (Δ = 3s); ReplayAccept: 0.000 (Δ = 1s) → 0.154 (Δ = 10s)	Choose Δ ≥ 3 s for high ASR with low replay acceptance
Nonce length |N| sensitivity (communication bits/device vs. nonce bytes) $\:(N=2000)$	20,880–21,264 bits/device (8–32B)	14,121–14,505 bits/device (8–32B)	≈ 30–34% reduction (stable)

Notes: Communication totals use the standardized 16-byte field model; computation timings use the calibrated unit costs (ECC ≈ 2.94 ms, hash ≈ 0.01 ms, XOR ≈ 0.001 ms, timestamp < 0.001 ms). The small ASR gap reflects the baseline’s repeated ECC verifies; SELAM attains near-baseline reliability with far lower overhead. Header-inclusive; *N* = 136 fixed UIDs; percentiles computed over successful authentications (completed Phases 1–6). Attack configuration: replay $\:{\boldsymbol{p}}_{\boldsymbol{r}}=0.05$ and impersonation $\:{\boldsymbol{p}}_{\boldsymbol{i}}=0.05$ with one extra injected attempt when triggered; statistics are aggregated over multiple RNG seeds (mean ± 95% CI). SELAM uses ECC only during provisioning (Phases A, B1, and 0). Heavy+Verify performs an online ECC verify in Phase 5.

## Security analysis

### Adversary model and security scope

We analyze SELAM under a standard Dolev–Yao network adversary for the online protocol phases. The adversary fully controls the communication channel between the user, medical device, gateway, and authentication server: it can eavesdrop, record transcripts, replay prior messages, delay or reorder traffic, drop messages, and inject fabricated messages, and it can initiate multiple concurrent sessions to attempt impersonation. The adversary is computationally bounded and does not break cryptographic primitives; the elliptic-curve discrete logarithm problem remains intractable for ECC instantiated with NIST P-256 (secp256r1), and the employed hash/HMAC functions are assumed to be collision-resistant and pseudorandom for authentication purposes. We assume the gateway and authentication server belong to the trusted computing base (i.e., are not compromised), clocks are loosely synchronized within a freshness window ± Δ used by the protocol, and side-channel leakage and physical device capture are out of scope. Under this model, SELAM targets mutual authentication, resistance to replay and impersonation attempts, and secrecy of the established session keys for completed sessions, while prioritizing minimal online asymmetric operations.

Table 8 summarizes SELAM’s security properties under assumptions A1–A5. Under this model, SELAM provides mutual authentication, replay/impersonation resistance, and explicit key confirmation via freshness enforcement (Δ), replay-cache control, and unforgeable authenticators (AEAD/HMAC) bound to the session transcript. Properties requiring stronger privacy primitives such as perfect forward secrecy and untraceability are not claimed in the baseline design and are listed as optional extensions (e.g., ephemeral ECDH or rotating pseudonyms with server-side mapping), consistent with SELAM’s design objective of eliminating online public-key operations.

Empirical protocol-level FAR and FRR are formally defined in Section V.E and computed from PhaseLogs. Both FAR and FRR are observed to be 0 under the evaluated adversarial conditions, supporting the robustness of SELAM within the stated assumptions.

**Table 8:** Security properties.

Security properties	Status	Mechanism in SELAM	Mitigation/ extension
Mutual authentication (user ↔ device / via GW/AS)	Provided (A1–A5)	Challenge–response integrity + credential binding; session accepted only if checks pass	–
Replay resistance	Provided (A2–A3)	Timestamp freshness (± Δ) + nonce/challenge binding prevents reuse of old transcripts	Tighten Δ; maintain lightweight replay cache at GW/AS
Impersonation resistance	Provided (A1–A4)	Forging valid responses requires secret material and valid integrity checks	Harden key storage: rate-limit failed attempts
Session key secrecy (established sessions)	Provided (A1, A3)	Session keys derived/confirmed only after successful integrity and freshness checks	Use key rotation for long-lived sessions
Key confirmation	Provided (A2–A3)	Protocol includes explicit confirmation step before session establishment	–
Perfect forward secrecy (PFS)	Not provided (baseline); optional extension	No per-session ephemeral ECDH is performed in online phases; compromise of long-term secrets may threaten past keys	Add optional ephemeral ECDH for high-risk sessions (cost-aware “upgrade” mode)
Anonymity / untraceability	Not claimed (baseline); optional extension	If stable identifiers appear on the channel, an eavesdropper can correlate sessions	Use rotating pseudonyms (hash-masked IDs refreshed per session) maintained by GW/AS
Traceability (revocable anonymity)	Not provided (baseline); optional with pseudonym refresh + server mapping	Baseline does not implement anonymity credentials; on-path identifiers remain linkable if stable	If pseudonyms are added, AS can maintain secure pseudonym → identity mapping
Non-repudiation	**Not provided**	Symmetric MAC/HMAC-style checks do not provide third-party verifiability	Add digital signatures where required (not lightweight)
DoS resilience	Partial (protocol-level only)	Early rejection on freshness/MAC failure; replay-cache drops; bounded retries	Rate limiting and filtering at GW; volumetric/network-layer DoS remains out of scope

A1: GW and AS are trusted (not compromised); device long-term secrets remain confidential.

A2: Freshness window Δ is enforced; replay cache prevents nonce reuse.

A3: AEAD provides confidentiality+integrity; HMAC is unforgeable; KDF is PRF-secure.

A4: Secure registration binds public keys/IDs to principals (Phases B1/0).

A5: Clocks are loosely synchronized within Δ.

We assume authenticated registration in Phases 0 and B1 (public keys bound to principals via a trusted authority or secure out-of-band channel), long-term seeds/keys remain confidential, clocks are synchronized within a freshness window Δ and the gateway prevents nonce reuse. In Phase 4 the gateway sends a session-key request authenticated via an AEAD tag under $\:{K}_{seed}\left({D}_{i}\right);\:$the device verifies the tag and freshness and both sides derive the same session key $\:{K}_{sess}\:\left({D}_{i}\right)=KDF\left({K}_{seed}\right({D}_{i})\parallel\:{ID}_{GW}\parallel\:{N}_{GW}\parallel\:{T}_{7}\text{}\:)$.

The device returns $\:{ACK}_{i}={HMAC}_{{K}_{seed}\left({D}_{i}\right)}({ID}_{{D}_{i}}\parallel\:{K}_{sess}\:({D}_{i})\parallel\:{T}_{7}$) which the gateway verifies, confirming shared-key installation. No online ECC is used.

We formally analyze Phases 4–5, the only online steps that establish authenticated beliefs and confirm a fresh session key. Phases B1/0 (registration) supply initial beliefs and the long-term seed $\:{K}_{\mathrm{seed}}\left({D}_{i}\right)$; Phase 1 is gateway access control; Phases 2–3 are control/trigger; Phase 6 merely consumes the established key. Phases 4–5 constitute the key-establishment and explicit-confirmation core and are analyzed with BAN logic. The remaining online phases (1–3, 6) are covered via structured lemmas under the same Dolev–Yao adversary model and standard cryptographic assumptions.

To address full online-path concerns, we (i) provide structured security reasoning for all online phases (Phases 1–6) under the Dolev–Yao network adversary and standard cryptographic assumptions (AEAD confidentiality/integrity; HMAC unforgeability), and (ii) give a BAN-logic derivation for Phases 4–5 where the fresh session key is established and confirmed. Earlier phases authenticate identities/credentials and bind freshness material (timestamps/nonces) to the session context, while Phase 6 only consumes the established session key.

### Phase-wise formal security argument (phases 1–3 and 6)

#### Lemma 1

**(Access-control authenticity and freshness).** A login/access request is accepted only if the gateway/authentication server validates a keyed authenticator bound to the claimed identity and verifies timestamp freshness within the window Δ. Under HMAC unforgeability (or AEAD integrity), an adversary without the corresponding secret cannot impersonate a legitimate principal; freshness checks reject delayed or replayed requests.

#### Lemma 2

**(Session binding and message integrity in Phases 2–3).** All initiation and request-submission messages carry an explicit session context (e.g., session identifier and/or step counter) and freshness material (timestamp and nonce). These values are included in the authenticated data / HMAC inputs, so message re-ordering, splicing across sessions, or transcript tampering is detected.

#### Lemma 3

**(Replay and reflection resistance).** Nonces are single-use (enforced via a replay cache), and timestamps must fall within Δ; hence, an attacker cannot successfully replay old transcripts or reflect challenges across sessions without violating freshness or authenticator checks.

#### Lemma 4

**(Secure-session confidentiality and integrity in Phase 6).** After Phase 5 confirms possession of the session key, subsequent application traffic is protected under the established session key using AEAD/HMAC. Confidentiality and integrity of protected payloads are reduced to AEAD security and session-key secrecy; any Man-in-the-Middle modification is detected by failed verification.

**Scope note.** This instantiation does not claim perfect forward secrecy (no online ephemeral key agreement), and privacy goals such as anonymity/untraceability are not provided unless rotating pseudonyms are added. Table 7 summarizes the provided and non-provided properties and practical mitigations.

### BAN logic correctness proof

We formalize the online part of SELAM with BAN logic^46^.

**Parties and preconditions.** The device $\:{D}_{i}$ and the gateway $\:GW$execute the online phases. The authentication server $\:AS$ provisions long-term material during registration and acts as an authority, while $\:GW$enforces freshness and nonce reuse checks.

**Goals.** Mutual authentication and key confirmation on a fresh $\:{K}_{\mathrm{sess}}\left({D}_{i}\right)$; informally, at completion, both $\:{D}_{i\:}$ and $\:GW\:$ believe they share a new session key bound to the current transcript.

**Idealized messages.**

$$\:\mathbf{M}1\::\:GW{\hspace{0.17em}}\to\:{\hspace{0.17em}}{D}_{i}:\{I{D}_{GW},{N}_{GW},{T}_{7}{\}}_{{K}_{\mathrm{seed}}\left({D}_{i}\right)}$$

$$\:\mathbf{M}2\::{D}_{i}\to\:GW:\{I{D}_{{D}_{i}},{K}_{\mathrm{sess}}({D}_{i}),{T}_{7}{\}}_{{K}_{\mathrm{seed}}\left({D}_{i}\right)}\mathrm{w}\mathrm{i}\mathrm{t}\mathrm{h}\:({\mathrm{A}\mathrm{C}\mathrm{K}}_{i}={\mathrm{H}\mathrm{M}\mathrm{A}\mathrm{C}}_{{K}_{\mathrm{seed}}\left({D}_{i}\right)}(I{D}_{{D}_{i}}\|\:{K}_{\mathrm{sess}}({D}_{i})\|\:{T}_{7}))$$

$$\:\mathbf{M}3\mathbf{a}\::\:GW\to\:{D}_{i}:\left\{{N}_{GW}^{{\prime\:}},{T}_{11},{\hspace{0.17em}}{\mathrm{m}\mathrm{a}\mathrm{c}}_{1}\right\}\text{}\mathrm{w}\mathrm{i}\mathrm{t}\mathrm{h}\:{\mathrm{m}\mathrm{a}\mathrm{c}}_{1}=h\left(\left({N}_{GW}^{{\prime\:}},{T}_{11}\right)\text{}\|{K}_{\mathrm{sess}}\left({D}_{i}\right)\right)$$

$$\:\mathbf{M}3\mathbf{b}\::\:{D}_{i}\to\:GW{:\{N}_{i}^{D},{T}_{11},{\hspace{0.17em}}{\mathrm{m}\mathrm{a}\mathrm{c}}_{2}\}\:\mathrm{w}\mathrm{i}\mathrm{t}\mathrm{h}\:{\mathrm{m}\mathrm{a}\mathrm{c}}_{2}=h\left(\left({N}_{GW}^{{\prime\:}}\oplus\:{N}_{i}^{D}\right)\text{}\|{K}_{\mathrm{sess}}\left({D}_{i}\right)\text{}\|{T}_{11}\right)$$

$$\:\mathbf{M}3\mathbf{c}\::GW\to\:{D}_{i}:\{{T}_{12},{\hspace{0.17em}}{\mathrm{m}\mathrm{a}\mathrm{c}}_{3}\}\:\mathrm{w}\mathrm{i}\mathrm{t}\mathrm{h}\:{\mathrm{m}\mathrm{a}\mathrm{c}}_{3}={\mathrm{H}\mathrm{M}\mathrm{A}\mathrm{C}}_{{K}_{\mathrm{sess}}\left({D}_{i}\right)}(I{D}_{{D}_{i}}\|\:I{D}_{GW}\| {N}_{i}^{D}\|\:{T}_{12})$$

**Beliefs and assumptions.**

A1.$\:{D}_{i}\mid\:\equiv\:GW\genfrac{}{}{0pt}{}{{K}_{\mathrm{seed}}\left({D}_{i}\right)}{\leftrightarrow\:}{D}_{i}$ and $\:GW\mid\:\equiv\:GW{\genfrac{}{}{0pt}{}{{K}_{\mathrm{seed}}\left({D}_{i}\right)}{\leftrightarrow\:}D}_{i}$ (shared long-term secret from registration).

A2. $\:{D}_{i}\mid\:\equiv\:\#({N}_{GW},{T}_{7})$ and GW$\:\mid\:\equiv\:\#({N}_{GW},{T}_{7})$; similarly, $\:\#\left({N}_{GW}^{{\prime\:}},{T}_{11},{T}_{12}\right)$ within a freshness window $\:{\Delta\:}$.

A3. $${D}_{i}|\:\equiv\:{K}_{\mathrm{sess}}\left({D}_{i}\right)=\mathrm{K}\mathrm{D}\mathrm{F}\left({K}_{\mathrm{seed}}\right({D}_{i}),I{D}_{GW},{N}_{GW},{T}_{7})\:\mathrm{a}\mathrm{n}\mathrm{d}\:GW\mid\:\equiv\:{K}_{\mathrm{sess}}\left({D}_{i}\right)=\mathrm{K}\mathrm{D}\mathrm{F}\left({K}_{\mathrm{seed}}\right({D}_{i}),I{D}_{GW},{N}_{GW},{T}_{7})$$  

(Jurisdiction: By A3 (KDF derivation rule), both parties derive $\:{K}_{sess}\left({D}_{i}\right)$ for this run.

A4. We model AEAD/HMAC tags as unforgeable authenticators: without the relevant secret key, an adversary cannot produce a valid tag on a new message or modify a protected message without detection (except negligible probability). Therefore, messages carrying valid tags are idealized as originating from the claimed principal who shares the corresponding key.

**Derivation using the three BAN inference rules.**

1. **Message-Meaning (MM) on M1.** From A1 and seeing $\:\{I{D}_{GW},{N}_{GW},{T}_{7}{\}}_{{K}_{\mathrm{seed}}}$:

$${\hspace{0.25em}\hspace{0.05em}}{D}_{i}\mid\:\equiv\:GW\mid\:\sim\:(I{D}_{GW},{N}_{GW},{T}_{7}).$$

2. **Nonce-Verification (NV) on M1.** With A2 freshness:
  $${\hspace{0.25em}\hspace{0.05em}}{D}_{i}\mid\:\equiv\:GW\mid \equiv\:\:(I{D}_{GW},{N}_{GW},{T}_{7}).$$
  By **Jurisdiction** (A3): ${D}_{i}\mid\:\equiv\:{K}_{\mathrm{sess}}\left({D}_{i}\right)$ is the intended fresh key for this run.

3. **Key derivation A3.**

$${D}_{i}\mid\:\equiv\:{K}_{\mathrm{sess}}\left({D}_{i}\right)=KDF\:\left(\cdot \right)\:and\:$$

$$\:GW\mid\:\equiv\:{K}_{\mathrm{sess}}\left({D}_{i}\right)=KDF\:\left(\cdot \right)$$

4. **Message-Meaning on M2 (ACK).** From A3′ (both know $\:{K}_{sess}$) and seeing $\:AC{K}_{i}$ under $\:{K}_{sess}$:$\mathrm{GW}\mid\:\equiv\:{D}_{i}\mid\:\sim\:(I{D}_{{D}_{i}},{K}_{\mathrm{sess}}({D}_{i}),{T}_{7}$  

5. **Nonce-Verification (NV) on M2.** With A2 freshness:

$$\mathrm{GW}\mid\:\equiv\:{D}_{i}|\:\equiv\:\left(I{D}_{{D}_{i}},{K}_{\mathrm{sess}}\left({D}_{i}\right),{T}_{7}\right)$$

This gives key confirmation from device to gateway.

6. **Phase-5 mutual confirmation (M3a–M3c)**:

Apply MM + NV similarly under $\:{K}_{sess}$ to conclude both sides believe the peer is live and the run is fresh (nonces/timestamps), hence mutual authentication is achieved for this transcript.

**Result.** By steps (1)–(5) using exactly Message-Meaning, Nonce-Verification, and Jurisdiction, SELAM achieves mutual authentication and key confirmation on a fresh $\:{K}_{\mathrm{sess}}\left({D}_{i}\right)\:$transcript-bound to $\:\left(I{D}_{GW},I{D}_{{D}_{i}},{N}_{GW},{T}_{7},{N}_{GW}^{{\prime\:}},{N}_{i}^{D},{T}_{11},{T}_{12}\right)$. Replay attempts are rejected by $\:{\Delta\:}$-freshness and transcript binding; impersonation requires forging AEAD/HMAC/keyed-hash tags under $\:{K}_{\mathrm{seed}}\left({D}_{i}\right)$or $\:{K}_{\mathrm{sess}}\left({D}_{i}\right)$; MitM alteration fails because any bit-level change invalidates the authenticators, and M3c provides explicit confirmation under $\:{K}_{\mathrm{sess}}\left({D}_{i}\right)$.

Replay is blocked by $\:{\Delta\:}$-fresh timestamps and nonces; impersonation and MitM fail under MAC unforgeability. Table 9 summarizes the properties established by the BAN-logic derivation for Phases 4–5 and the corresponding mechanisms in SELAM.

**Table 9:** Security mechanisms and phase mapping in SELAM.

Goal/attack	Mechanism	Phases (steps)	Outcome
Mutual authentication & key confirmation	Phase-4 ACK under $\:{K}_{seed}\left({D}_{i}\right)$; Phase-5 challenge–response and confirmation under $\:{K}_{sees}\left({D}_{i}\right)$	4 (ACK); 5 (1–8)	Achieved
Replay	Timestamp window Δ + one-time nonces on all authenticated tokens	0, B1, 4, 5	Prevented
Impersonation	Only holder of $\:{K}_{seed}\left({D}_{i}\right)\:($or derived $\:{K}_{sees}\left({D}_{i}\right)$) can produce valid HMACs	B1, 4, 5	Prevented
MitM	Phase-4 AEAD-authenticated session-key package (IDs/counters/timestamp as AD); Phase-5 HMAC-authenticated security-critical fields	4, 5	Prevented *(under freshness and MAC unforgeability)*
Transcript Integrity & session binding	HMAC over IDs, nonces, timestamp, step context in Phase-5 binds the transcript to this run	5	Ensured

### Security properties beyond mutual authentication

SELAM provides mutual authentication and key confirmation for online sessions and blocks replay under the freshness window $\:{\Delta\:}$ and single-use nonces (enforced by a replay cache). However, because SELAM does not run a fresh ECDH exchange in the online path, it does not provide perfect forward secrecy (PFS): compromise of a long-term device secret can enable an adversary to recompute past and future session keys derived from that secret (i.e., no PFS). SELAM also does not claim full anonymity or untraceability: device identifiers are pseudonymous but linkable if they remain stable within a deployment, so a network observer may correlate repeated authentications to the same device. SELAM instead prioritizes integrity, replay resistance, and lightweight mutual authentication under the stated Dolev–Yao model. If stronger privacy is required, SELAM can be combined with periodic pseudonym refresh or identifier-hiding tokens derived from a server-held secret, at the cost of additional state and/or communication. These privacy extensions are orthogonal to SELAM’s primary design point, which is to eliminate online public-key operations for critical-device authentication.

#### Scope and limitations of formal verification

While BAN logic provides formal validation for mutual authentication and key confirmation in Phases 4–5, the remaining online phases are analyzed using structured security reasoning under the Dolev–Yao adversary model. Although automated formal verification tools such as ProVerif and Tamarin can offer stronger guarantees by exhaustively analyzing protocol behaviors, their application to the full online authentication pipeline (Phases 1–6) is beyond the scope of this work. This study instead emphasizes lightweight protocol design and large-scale empirical validation under adversarial conditions. Extending the analysis to automated formal verification frameworks remains an important direction for future work.

## Conclusion and future works

This paper presented SELAM, a selective-ECC lightweight authentication framework for IoMT in which public-key operations are confined to one-time user/device registration. In contrast, online authentication relies on hash/HMAC, XOR, and timestamp-freshness validation. A KDF-based derivation in Phase 4 establishes the session key without per-message ECC, and Phase 5 provides explicit key confirmation via lightweight challenge–response. We formalized the protocol phases (B1, 0, 1–6), aligned the notation with the system model, and derived analytical expressions for communication, computation, and end-to-end authentication overhead.

Under standardized 16-byte online field accounting, SELAM reduces per-round communication from 960 B to 768 B (≈ 20%) compared to a Heavy+Verify ECC baseline. Eliminating online ECC reduces per-device computation from ≈ 23.59 ms to ≈ 0.10 ms (≈ 99.6%), yielding a total per-device authentication overhead of ≈ 6.24 ms at 1 Mb/s, compared to ≈ 31.27 ms for the baseline. Dataset-driven ns-3 experiments corroborate the analysis: SELAM’s savings arise in the online key-establishment/confirmation path, while maintaining attack-regime ASR at ≈ 0.88–0.90, within ~ 2–3% of the baseline across $\:N=136$–$\:2000$ over 20 seeds (mean ± 95% CI). Protocol-level PhaseLog analysis further shows FAR=0 (no accepted replay/impersonation) and benign FRR=0 in the evaluated settings. Overall, SELAM demonstrates that selective ECC can preserve strong authentication guarantees while removing public-key operations from the performance-critical online path for resource-constrained critical medical devices.

### Limitations and outlook

The present study intentionally scopes SELAM to single-device authentication with amortized registration and single-hop delivery; clustered/group authentication, multi-hop propagation effects, and mobility-driven dynamics are not modeled. Because SELAM does not perform per-session ECDH and restricts asymmetric cryptography to setup/registration, perfect forward secrecy (PFS) is not provided, and the scheme does not claim key-compromise impersonation (KCI) resistance if a device’s long-term seed is exposed. Security validation includes BAN-logic analysis for the session-key establishment phases, while automated formal verification (e.g., ProVerif/Tamarin) for the full authentication pipeline is outside the scope of this work. In addition, physical side-channel resistance is not considered, as this study focuses on protocol-level security rather than hardware-level attack surfaces. Finally, denial-of-service is assessed only at the protocol level via elevated authentication request rates; volumetric or network-layer DoS remains out of scope.

Future work will extend SELAM along three directions:

(i) scalability through clustered deployment and batch processing, measuring how gateway aggregation and group session-key management reduce authentication delay and communication overhead as the number of devices grows;

(ii) extension of adversarial evaluation beyond the current replay, impersonation, and man-in-the-middle settings, including higher-intensity attack traffic and more heterogeneous operational conditions, by analyzing authentication success ratio, processing delay, and communication cost; and (iii) automated verification using formal tools (e.g., ProVerif or Tamarin) together with targeted side-channel considerations. These extensions will provide a more comprehensive validation of SELAM in practical, large-scale healthcare environments.

## Data Availability

The data supporting the findings of this study are available from the corresponding author upon request.
